# DEPS-1 is required for piRNA-dependent silencing and PIWI condensate organisation in *Caenorhabditis elegans*

**DOI:** 10.1038/s41467-020-18089-1

**Published:** 2020-08-25

**Authors:** Kin Man Suen, Fabian Braukmann, Richard Butler, Dalila Bensaddek, Alper Akay, Chi-Chuan Lin, Dovilė Milonaitytė, Neel Doshi, Alexandra Sapetschnig, Angus Lamond, John Edward Ladbury, Eric Alexander Miska

**Affiliations:** 1 Wellcome Trust Cancer Research UK Gurdon Institute, University of Cambridge, Tennis Court Road, Cambridge, CB2 1QN UK; 2 Department of Genetics, University of Cambridge, Downing Street, Cambridge, CB2 3EH UK; 3 Laboratory for Quantitative Proteomics, Centre for Gene Regulation and Expression, College of Life Sciences, University of Dundee, Dow Street, Dundee, DD1 5EH UK; 4 School of Molecular and Cellular Biology, University of Leeds, LC Miall Building, Leeds, LS2 9JT UK; 5 University of Cambridge, School of Clinical Medicine, Cambridge Biomedical Campus, Cambridge, CB2 0SP UK; 6 Wellcome Sanger Institute, Wellcome Trust Genome Campus, Hinxton, CB10 1SA UK; 7 Present Address: Bioscience Core labs, King Abdullah University of Science and Technology, Thuwal, 23955-6900 Saudi Arabia; 8 Present Address: School of Biological Sciences, University of East Anglia, Norwich, NR4 7TJ UK

**Keywords:** Epigenetic memory, Gene silencing, RNAi, Piwi RNAs

## Abstract

Membraneless organelles are sites for RNA biology including small non-coding RNA (ncRNA) mediated gene silencing. How small ncRNAs utilise phase separated environments for their function is unclear. We investigated how the PIWI-interacting RNA (piRNA) pathway engages with the membraneless organelle P granule in *Caenorhabditis elegans*. Proteomic analysis of the PIWI protein PRG-1 reveals an interaction with the constitutive P granule protein DEPS-1. DEPS-1 is not required for piRNA biogenesis but piRNA-dependent silencing: *deps-1* mutants fail to produce the secondary endo-siRNAs required for the silencing of piRNA targets. We identify a motif on DEPS-1 which mediates a direct interaction with PRG-1. DEPS-1 and PRG-1 form intertwining clusters to build elongated condensates in vivo which are dependent on the Piwi-interacting motif of DEPS-1. Additionally, we identify EDG-1 as an interactor of DEPS-1 and PRG-1. Our study reveals how specific protein-protein interactions drive the spatial organisation and piRNA-dependent silencing within membraneless organelles.

## Abstract (web summary)

The *C. elegans* PIWI protein PRG-1 resides in the membraneless organelle P granule. Here, the authors identify the constitutive P granule protein DEPS-1 as an interactor of PRG-1 and show its function in piRNA-dependent silencing. DEPS-1 and PRG-1 form elongated condensates in vivo.

## Introduction

The correct spatial organisation of molecules into organelles is essential for biological function. Recent studies reveal that membraneless organelles can be formed by proteins and nucleic acids condensing out of the bulk intracellular milieu, giving rise to liquid- or gel-like environments^1–3^. These phase separated organelles, formed by proteins and nucleic acids, are sites for different aspects of eukaryotic RNA biology: the nucleolus is required for the assembly of ribosomes^4^, stress granules allow for translational stalling of mRNAs during stress-response^5^ and processing bodies (P-bodies) organise small RNA-mediated regulation of mRNA^6^. However, the molecular mechanisms of how RNAs and proteins assemble into phase separated organelles remain largely unexplored.

Small non-coding RNAs execute diverse biological functions. mRNAs are targeted for silencing by small RNAs based on Watson-Crick base-pair complementarity in complex with members of the Argonaute (Ago) protein family^7^. Various Ago proteins associate with membraneless organelles such as the P-bodies and germ granules^8,9^. Indeed, recently it has been shown that human Ago2 together with its binding partner TNRC6B can form biomolecular condensates entirely on their own^10^. A recently discovered condensate, the Z granule, contains the Ago protein WAGO-4 to establish transgenerational inheritance (TEI) of RNAi in *C. elegans*^11,12^. Hence, some small RNAs are routed through membraneless organelles. To further our understanding of how small RNAs operate within membraneless organelles, we turn to the piRNA pathway.

piRNAs associate with the PIWI clade proteins in the Argonaute family to repress transposable elements (TEs)^13–15^. Mutations in the piRNA pathway lead to varying degrees of infertility, indicating it plays an essential role in the survival of a species. For example, null mutations in each of the three *piwi*-coding genes lead to sterility in male mice^16–18^; depletion of the single functional PIWI protein in *C. elegans* leads to reduced fecundity^19^; in humans, a mutation blocking the ubiquitination of the PIWI protein HIWI has been implicated in azoospermia^20^.

Mature piRNAs mediate transcriptional and post-transcriptional gene silencing. In *C. elegans*, piRNAs are 21 nt long with a 5′ preference for U^21^ and contain 2-O-methylation at the 3′ end^22,23^. piRNAs associate with the PIWI protein PRG-1 to scan for target mRNAs(21Us)^24,25^. These target mRNAs then serve as templates for the production of endo-siRNAs that are 22 nt long with a 5′ preference for G (22Gs) by the RNA-dependent RNA polymerases (RdRPs) EGO-1 and RRF-1^25–28^. The *C. elegans* piRNA pathway offers a unique model for understanding how membraneless organelles engage with small RNA pathways as it requires proteins that can localise to two distinct and juxtaposed biomolecular condensates to achieve gene repression: the perinuclear P granules where PRG-1 resides^24^ and the secondary endo-siRNAs are entirely dependent on the mutator foci^29–31^.

In this work, we investigate how the piRNA pathway engages with the membraneless organelles for its function in *C. elegans*. First, we determine the protein interactome of the piRNA-binding protein PRG-1 and show a direct interaction with DEPS-1, a protein that associates with the P granule. We also identify the PRG-1-binding domain on DEPS-1 and show that this domain is required for silencing by piRNA, and for the typical morphology of PRG-1 condensates. We show that functionally, *deps-1* and the interaction between DEPS-1 and PRG1 are required for the steady state levels of secondary endo-siRNAs. Hence, DEPS-1 functions as a bridge between piRNAs and secondary endo-siRNAs. Our study reveals that small RNA pathways and membraneless organelles are interdependent and that an essential P granule factor actively participates in small RNA regulation by directly binding to an Argonaute protein.

## Results

### DEPS-1 forms a protein complex with PRG-1

To identify proteins that intersect biomolecular condensate functions and small RNA pathways we performed immunoprecipitation (IP) of PRG-1 followed by mass spectrometry (Supplementary Fig. S1a). This led us to identify 133 proteins to be preferentially in a complex with PRG-1 (Supplementary Data 1). Of the 133 putative PRG-1 interactors, the P granule factor DEPS-1 (Defective P granules and Sterile-1) was identified as a binding candidate and was among the ten most enriched factors (Supplementary Fig. S1b, c, d). DEPS-1 is a constitutive member of P granules and required for the correct assembly of P granules^32^ as well as for transgenerational inheritance (TEI) of exogenous RNAi^11,33^. It is not predicted to contain any known domain folds but consists of a poly-serine C-terminal end. Prediction for secondary structures suggests the protein is composed mainly of beta-sheets and loops (Supplementary Fig. S1e). As P granule formation depends on self-interaction domains in its constituent proteins, many proteins required for P granule formation contain domains with long stretches of low complexity in amino acid composition^3^. However, DEPS-1 is not predicted to possess such domains despite its requirement for P granule integrity (Supplementary Fig. S1f).

### DEPS-1 and PRG-1 form intertwining clusters

**Fig. 1:**
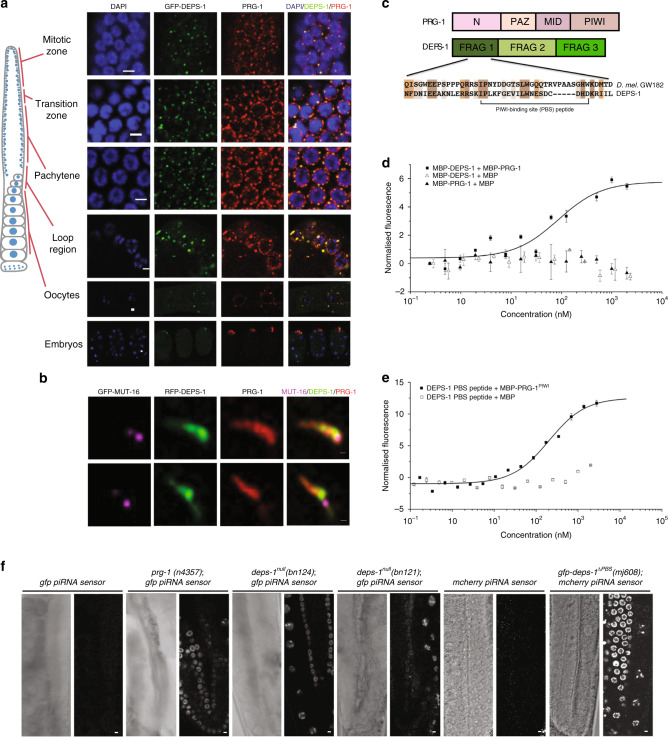
DEPS-1 binds to PRG-1 and mediates piRNA-dependent transgene silencing. **a** DEPS-1 and PRG-1 colocalise as peri-nuclear granules. *C. elegans* germlines expressing GFP-DEPS-1 were dissected and immunostained for GFP and PRG-1. PRG-1 and DEPS-1 colocalise in the proliferative zone, transition zone, pachytene, oocytes and embryos. A higher proportion of PRG-1 remains perinuclear in the loop region compared to DEPS-1. Scale bar = 3 µm. **b** Colocalisation of PRG-1, DEPS-1 and MUT-16. Dissected germlines co-stained for PRG-1, RFP-DEPS-1 and GFP-MUT-16 were imaged and deconvoluted with HyVolution settings. Two clusters with all three proteins present are shown. Scale bar = 0.25 µm. **c** Domain/fragment architecture of PRG-1 and DEPS-1. Sequence alignment (Clustal W) of Ago binding motif II on *Drosophila melanogaster* GW182 and DEPS-1 PBS with flanking sequences. **d** Recombinant full-length MBP-tagged PRG-1 and MBP-tagged DEPS-1 were purified for MST assays. A serial dilution of unlabelled MBP-PRG-1 was incubated with 10 nM of label MBP-DEPS-1 and tested for binding. The *K*_d_^app^ is 855 ± 133 nM. Representative of *n* = 2 independent experiments. Data are presented as mean values ± SD of three technical replicates. Black square = MBP-DEPS-1 and MBP-PRG-1, black triangle = MBP-PRG and MBP, open triangle = MBP-DEPS-1 and MBP titrations. **e** MST measurement of fluorescently labelled DEPS-1 PBS motif peptide was incubated with unlabelled MBP-tagged PRG-1 PIWI domain. *K*_d_^app^ is 1.9 µM ± 98 nM. Representative of n=2 independent experiments. Data are presented as mean values ± SD of 3 technical replicates. Black square = DEPS-1 PBS peptide and MBP-PRG-1^PIWI^, open square = DEPS-1 PBS peptide and MBP titrations. **f** Mutations in *deps-1* lead to piRNA sensor transgene desilencing. Whitefield (right panel) and fluorescent (left panel) images of whole mounted animals show the piRNA sensor is efficiently silenced in wild-type animals and is desilenced in *prg-1(n4357), deps-1*^*null*^ *(bn121 and bn124)* and *deps-1*^*∆PBS*^*::rfp(mj608)* mutants. Scale bar = 3 µm.

To further study the interaction between PRG-1 and DEPS-1, we asked whether the proteins colocalise in vivo. First, we confirmed that colocalise in P granules by co-immunostaining transgenic animals expressing GFP-DEPS-1^34^ (Fig. 1a). In the adult germline, both proteins colocalise to P granules from the mitotic zone to the pachytene region. In the distal loop region, where oogenesis begins and P granules start to disperse from the nuclear membrane, a higher proportion of GFP-DEPS-1 starts to dissociate from the perinuclear region than PRG-1, suggesting the proteins are differentially regulated during a small temporal window. Given that PRG-1 binds to piRNAs to trigger secondary endo-siRNA biogenesis, we asked if DEPS-1 complexes colocalise with the mutator foci, which are biomolecular condensates that house essential endo-siRNA factors. While at lower resolution PRG-1 as well as DEPS-1 appear to be condensates that overlap with each other, these condensates can be further resolved to clusters of proteins at higher resolution^35^ in the pachytene region (Fig. 1b). Often these DEPS-1 and PRG-1 clusters weave around each other to form elongated condensates. In contrast, MUT-16 condensates do not resolve to smaller clusters and only juxtaposed close to the DEPS-1/PRG-1 complex (Fig. 1b), consistent with previous findings^30^. We investigated how DEPS-1 and PRG-1 clusters are positioned relative to the P granule protein PGL-1 in the pachytene region. We found that PRG-1 and PGL-1 form intertwining clusters while DEPS-1 and PGL-1 clusters overlap with each other (Supplementary Fig. S1g). ZNFX-1 forms condensates closely appose to PGL-1 and MUT-16^11^. ZNFX-1 is also found in close proximity to DEPS-1 clusters, consistent with DEPS-1 colocalisation with PGL-1 (Supplementary Fig. S1h).

### DEPS-1 binds to PRG-1 via its PIWI Binding Site (PBS)

To investigate if the DEPS-1/PRG-1 interaction is direct and RNA-independent, we purified recombinant RNA-free full-length DEPS-1 and PRG-1 as MBP-fusion proteins and tested for binding using microscale thermophoresis (MST). DEPS-1 and PRG-1 interact with *K*_d_^app^ at 855 ± 133 nM (Fig. 1c, d and Supplementary Fig. S2a). So far only a few examples of direct interactors for the Ago protein family have been identified, in particular for the PIWI clade. A sub-micromolar dissociation constant is on par with GW182 and Tudor domain-containing proteins interaction with human Ago and mouse PIWI^36,37^ suggesting DEPS-1 and PRG-1 binding is physiologically relevant.

To dissect the nature of the interaction, we truncated PRG-1 to its individual domains (Fig. 1c) and identified the PIWI domain (PRG-1^PIWI^) to be responsible for binding to full-length DEPS-1 using MST (Supplementary Fig. S2b, c). We were unable to rule out DEPS-1 interacting with the MID domain as the MBP-tagged MID domain of PRG-1 binds to the MBP tag non-specifically (Supplementary Fig. S2d). PRG-1^PIWI^ binds to full length DEPS-1 with *K*_d_^app^ = 349 ± 45 nM which is comparable to the interactions with full-length PRG-1 suggesting that PRG-1 interacts with DEPS-1 with its PIWI domain.

To identify which region of DEPS-1 is required for binding to PRG-1, we truncated DEPS-1 into three fragments of similar sizes and with fragment boundaries in regions lacking predicted secondary structures (Fig. 1c). We detected binding between PRG-1^PIWI^ and the N-terminal fragment of DEPS-1 (DEPS-1^frag1^) only with a *K*_d_^app^ of 151 ± 28 nM (Supplementary Fig. S2e, f). This is again in agreement with the binding between the full-length proteins indicating that DEPS-1 interacts with its N-terminal region with PRG-1 PIWI domain.

Given that DEPS-1 interacts with PRG-1 via PRG-1^PIWI^ and that the PIWI domains of PIWI and Ago families share similar folds overall^37^, we next asked if DEPS-1 shares any characteristics with known protein interactors of the Ago PIWI domain. The GW182 proteins have been shown to bind to the Ago PIWI domain by its multiple GW motifs which fit into tryptophan-binding pockets^10,38,39^. While DEPS-1 lacks any GW motifs, we noticed a degree of similarity between two short stretches of DEPS-1 and the *D. melanogaster* GW182 in our alignment (GW182), one at the N-terminal and the other the C-terminal of DEPS-1. While the C-terminal region with similarity to dmGW182 is the poly-serine tail, the N-terminal region of DEPS-1 shares similarity with dmGW192’s Ago-binding motif II^36,40^ (Fig. 1c). Moreover, this N-terminal region is contained within DEPS-1^frag1^ which binds to PRG-1^PIWI^. We therefore generated a peptide for part of this sequence (DEPS-1^peptide^; Fig. 1c) to test its binding with PRG-1^PIWI^. DEPS-1^peptide^ binds to PRG-1^PIWI^ with a K_d_^app^ of 1.9 ± 0.1 µM indicating this Ago-binding motif II-like region of DEPS-1 is indeed responsible for PRG-1 interaction (Fig. 1e). We have termed this motif the PIWI-binding site (PBS). Upon removal of the PBS, PRG-1^PIWI^ fails to bind to DEPS-1^frag1^ (Supplementary Fig. 2g).

### DEPS-1 is required for piRNA-dependent silencing

Using the piRNA sensor, we next asked if *deps-1* functions in the piRNA pathway in vivo. The piRNA sensor is a genetic tool consisting of a GFP- or mcherry- tagged histone 2B (H2B) with a piRNA target site at its 3′ end, rendering its expression dependent on the piRNA pathway^25^. We analysed the effect of the PRG-1/DEPS-1 binding by removing the PBS from endogenous *deps-1* and replacing it with a 5x glycine residue-linker via CRISPR-Cas9 gene editing (henceforth referred to as *deps-1*^*∆PBS*^*)* as well as two *deps-1* null alleles (*bn121* and *bn124*)^32^. Crossing *deps-*1 mutants with piRNA sensor animals, we found that the piRNA sensor is de-silenced in both *deps-1*^*null*^ mutants, as well as the *deps-1*^*∆PBS*^ mutant, as in the *prg-1(n4357)* mutant, indicating that *deps-1* and specifically its PBS is required for piRNA-dependent silencing (Fig. 1f, Supplementary Fig. S3a). Correspondingly, small RNAs targeting the piRNA sensor are reduced in *deps-1*^*null*^ mutants (Supplementary Fig. S3b). Hence, DEPS-1 binding to PRG-1 is required for normal piRNA pathway activity.

Having seen that *deps-1*^*∆PBS*^ has a similar effect on piRNA function as *deps-1* null mutation, we tested if *deps-1*^*∆PBS*^ is also resistant to germline RNAi as observed in the *deps-1* null mutants^32^. Knockdown of *pos-1* results in dead embryos. Indeed, *deps-1*^*∆PBS*^ is resistant to RNAi in the germline (Supplementary Fig. S3c).

### PRG-1 condensate organisation needs PRG-1 and DEPS-1 binding

**Fig. 2:**
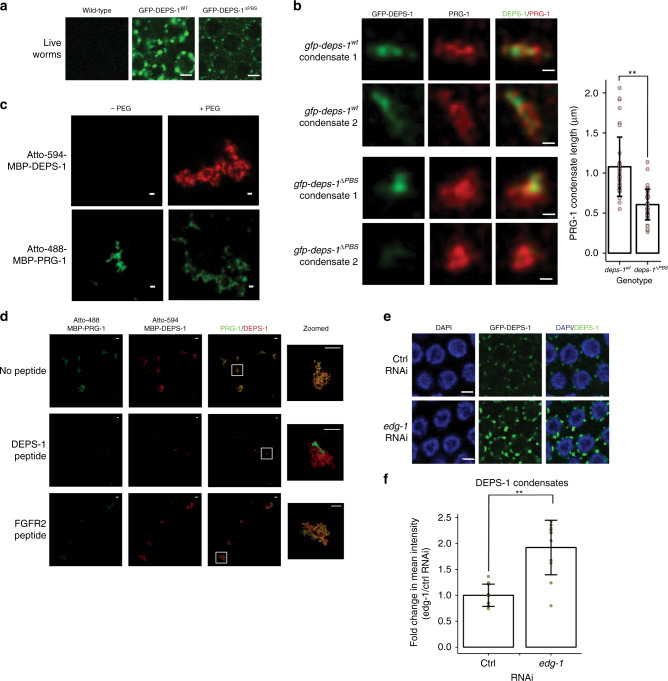
PRG-1 and DEPS-1 intertwining organisation is dependent on PBS on DEPS-1. **a** Live worm imaging of the germline of wild-type animals (N2) (left), animals expressing wild-type GFP-DEPS-1 (*ax2063*; GFP-DEPS-1^WT^; middle) or GFP-DEPS-1 with mutated PBS (*mj608;* GFP-DEPS-1^∆PBS^; right). GFP-DEPS-1^∆PBS^ forms fewer granules and instead is diffused in the cytoplasm compared with GFP-DEPS-1^WT^. Scale bar = 3 µm. **b** DEPS-1 and PRG-1 condensates are malformed in *gfp::deps-1*^*∆PBS*^*(mj608)* mutant. Dissected germlines co-stained for PRG-1 and GFP-DEPS-1 were imaged and deconvoluted with Hyvolution settings. Two selected clusters of each genotype were enlarged to show differences between the wild-type and PBS mutant form of GFP-DEPS-1. Top panels: *gfp::deps-1*^*WT*^*(ax2063)*; Bottom panels: *gfp::deps-1*^*∆PBS*^*(mj608)*. The length of PRG-1 condensate along the perinuclear membrane was measured manually. Bar graph represents twenty PRG-1 condensates measured in two germlines (total *n* = 40 for each genotype). Scale bar = 0.25 µm. Data represented here as mean values ± SD. Kolmogorov–Smirnov test was performed, **p value < 0.005. Source data are provided as a Source Data file. **c** Recombinant DEPS-1 and PRG-1 form small clusters similar to in vivo proteins. 1.7 µM MBP-tagged DEPS-1 (top panel) and 0.6 µM MBP-tagged PRG-1 were labelled with Atto-488 or −594, respectively. While DEPS-1 was only able to form small protein clusters in the presence of 5% PEG2000, PRG-1 formed clusters even in absence of PEG2000. Scale bar = 0.5 µm. **d** Intertwining clusters of recombinant DEPS-1 and PRG-1 is dependent on the PBS motif on DEPS-1. 3.4 µM Atto-488 labelled MBP-tagged DEPS-1 was incubated with 0.6 µM Atto-594 labelled MBP-tagged PRG-1 in the presence of 5% PEG2000. The two protein clusters associate with each other. When PRG-1 was preincubated with 2 mM of a peptide containing the DEPS-1 PBS motif, the two proteins failed to co-localise. An unrelated peptide (2 mM FGFR2 peptide) does not disrupt DEPS-1 and PRG-1 clusters association. Scale bar = 5 µm. **e** *edg-1* was knocked down via RNAi in animals expressing *deps-1::gfp(ax2063)*. Animals were dissected for germline staining of GFP in the pachytene region. DEPS-1 forms brighter granules upon *edg-1* knockdown. Scale bar = 3 µm. **f** DEPS-1 condensates are more intense upon RNAi knockdown of *edg-1*. *n* = 10 per genotype. Data represented here as mean values ± SD. Two-sided *t*-tests were performed (**p value < 0.005). Source data are provided as a Source Data file.

Having identified the PRG-1 binding site on DEPS-1 and shown that it is required for piRNA-dependent silencing, we asked how the removal of this site affects DEPS-1 localisation. Live imaging of GFP-DEPS-1^∆PBS^ expressing animals in the pachytene region revealed that in the absence of PBS, DEPS-1 becomes diffused in the cytoplasm and forms fewer granules (Fig. 2a, Supplementary Fig. S3d; expression of GFP-DEPS-1^∆PBS^ is at ~70% of the wild-type protein). We imaged the condensates at a high resolution to inspect how the DEPS-1 and PRG-1 cluster organisation is affected. As shown before wild-type DEPS-1 and PRG-1 clusters intertwine each other to form elongated condensates. While DEPS-1^∆PBS^ localises to PRG-1 condensates when DEPS-1 is able to associate with the peri-nuclear region, PRG-1 condensates contain either very little or no DEPS-1^∆PBS^ (Supplementary Fig. S3e). Furthermore, PRG-1 clusters do not intertwine with DEPS-1^∆PBS^. We measured the length of the PRG-1 condensates along the peri-nuclear edge and found that PRG-1 condensates (with and without DEPS-1^∆PBS^ in *deps-1*^*∆PBS*^ animals and *deps-1* null animals) become more compacted compared with the PRG-1/DEPS-1^WT^ elongated condensates (Fig. 2b, Supplementary Fig. S3f, g). Hence, the peri-nuclear organisation of PRG-1 clusters is maintained by its direct interaction with DEPS-1.

### PRG-1 and DEPS-1 intrinsically form clusters in vitro

We wondered if the ability to form these small clusters of DEPS-1 and PRG-1 is intrinsic to these proteins. We fluorescently labelled recombinant MBP-tagged DEPS-1 and PRG-1 full length proteins for high-resolution confocal imaging. We found that PRG-1, but not DEPS-1, was able to form small clusters (Fig. 2c). To mimic the crowded environment of the P granule^41,42^, we incubated the proteins with 5% PEG2000. In the presence of the molecular crowding agent, DEPS-1 are able to form clusters. Furthermore, these in vitro clusters are of similar size to the in vivo clusters (~250 nm in diameter). This suggests the formation of these sub-organelle clusters are intrinsic to the protein sequences of DEPS-1 and PRG-1. We incubated the two proteins together in the presence of PEG2000 to see if they form intertwining clusters similar to those observed in vivo. Indeed, the DEPS-1 and PRG-1 clusters associate with each other (Fig. 2d, Supplementary Fig. S3h). However, they do not form the elongated structures observed in vivo, indicating the elongation is dependent on interactions with other intracellular components. Finally, the association between DEPS-1 and PRG-1 clusters can be disrupted by the presence of DEPS-1^PEPTIDE^ but not by the addition of an unrelated, human FGFR2 peptide, which is unable to compete with DEPS-1 for binding to PRG-1 (Fig. 2d). Hence, DEPS-1 and PRG-1 clusters association requires the PBS motif on DEPS-1.

### EDG-1 binds DEPS-1 and PRG-1 and modifies DEPS-1 condensates

Despite the uncoupling of DEPS-1 from PRG-1 by the deletion of PBS, DEPS-1^∆PBS^ and PRG-1 remain in the same P granules at a low level. We therefore wondered if other proteins form a complex with them. We performed a yeast-two-hybrid (Y2H) screen using full length DEPS-1 as a bait. We obtained one high-confidence candidate - the putative protein encoded by *B0035.6*. Y2H data indicated that *B0035.6* interacts with DEPS-1 via its C-terminal region (Supplementary Fig. S4a). B0035.6 has no predicted conserved domain structures but a low similarity to human MEG-3^43^. B0035.6 was also one of the significant interactors of PRG-1 in our proteomic analysis (Supplementary Data 1 and Supplementary Fig. S4b), suggesting DEPS-1, B0035.6 and PRG-1 form a trimeric complex. We then performed RNAi knock-down of *B0035.6* and tested if it is required for the normal formation of DEPS-1 or PRG-1 condensates. Reducing B0035.6 expression level lead to enlarged DEPS-1 condensates (Fig. 2e, f), but not PRG-1 condensates nor the P granule protein PGL-1 (Supplementary Fig. S4c, d, e). We therefore named *B0035.6* as *Enlarged* *Deps* *Granules-1 (edg-1)*. Given the dependence of the piRNA pathway on the mutator foci, we tested if MUT-16 condensation was affected and found that they are not (Supplementary Fig. S4c). Hence, while *edg-1* is found to be an interactor for both DEPS-1 and PRG-1, it specifically modulates DEPS-1 condensation. However, it is unclear whether the change in DEPS-1 condensate mediated by *edg-1* knockdown is due to a change in DEPS-1 protein level.

### DEPS-1, PRG-1 and mutator condensates are interdependent

**Fig. 3:**
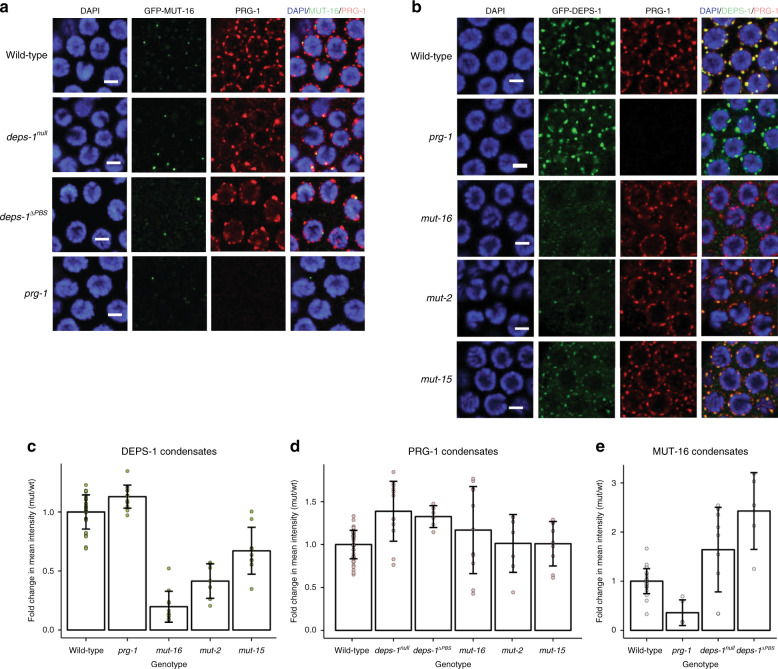
Morphologies of PRG-1, DEPS-1 and MUT-16 condensates are interdependent. **a** GFP-MUT-16 and PRG-1 localisations were examined in *prg-1(n4357), deps-1*^*null*^*(bn121) and deps-1*^*ΔPBS*^*(mj605)* mutants. The common genotypes of strains used are *mut-16(pk710); gfp::mut-16(mgSi2)* which is denoted as ‘wild-type’. Additional mutations upon this common genotype are indicated on the left. *C. elegans* germlines were dissected and immunostained for GFP and PRG-1. Scale bar = 3 µm. **b** GFP-DEPS-1 and PRG-1 localisations were examined in *prg-1(n4357)*, *mut-16(pk710), mut-2(ne298) and mut-15(tm1358)* mutants. ‘wild-type’ indicates the common genotype of *gfp::deps-1(ax2063)* among the strains used and additional mutations are indicated on the left. *C. elegans* germlines were dissected and immunostained for GFP and PRG-1. Scale bar = 3 µm. **c** Fold change in DEPS-1 condensate density in wild-type (n = 41), *prg-1* (*n* = 13), *mut-16* (*n* = 11), *mut-2* (*n* = 7), *mut-15* (*n* = 9) animals. **d** Fold change in PRG-1 condensate density in wild-type (*n* = 37), *mut-15* (*n* = 9), *mut-16* (*n* = 11), *deps-1*^*null*^ (*n* = 12), *deps-1*^*ΔPBS*^ (*n* = 3), *mut-2* (*n* = 7) animals. **e** Fold change in MUT-16 condensate density in wild-type (n = 25), *prg-1* (*n* = 5), *deps-1*^*null*^ (*n* = 9), *deps-1*^*ΔPBS*^ (*n* = 5) animals. **c**–**e** Data are presented as mean values ± SD. Source data are provided as a Source Data file.

piRNA function requires protein factors in the P granule as well as the mutator foci; we investigated how DEPS-1, PRG-1 and MUT-16 condensates are affected by mutations in *deps-1*, *prg-1* and *mutator* genes. In the pachytene region, *deps-1*^*null*^ and *deps-1*^*∆PBS*^ mutations lead to PRG-1 condensates becoming brighter as reflected by higher condensate intensity, suggesting either more proteins are present in the condensates or PRG-1 becomes more densely packed (Fig. 3a, d and Table 1). Since *deps-1* mutations have been shown to alter the levels of the mRNA and proteins of P granule factors^32^, we investigated if *deps-1* mutations also affect *prg-1*. No significant differences in either mRNA or protein products of *prg-1* in *deps-1* mutants were detected (Supplementary Fig. S5a, b, c). Hence, the effects of *deps-1* on PRG-1 condensate are solely in the subcellular distribution of the protein. Intriguingly, *deps-1* mutants contain fewer and brighter MUT-16 condensates despite DEPS-1 being a P granule protein (Fig. 3a, Table 1 and Supplementary Fig. S5d). However, whether the change in MUT-16 condensates is due to protein expression level or localisation defects remains to be determined.

**Table 1:** Condensate defects in *prg-1, deps-1* and *mutator* foci mutants.

Control strain genotype	Test strain genotype	Relevant mutation	Condensate	Intensity	Area	Circularity
*deps-1::gfp(ax2063)*	*gfp::deps-1(ax2063); mut-15(tm1358)*	*mut-15*	GFP-DEPS-1	++^down^	+++ ^down^	++^up^
*deps-1::gfp(ax2063)*	*gfp::deps-1(ax2063); mut-16(pk710)*	*mut-16*	GFP-DEPS-1	+++^down;^*	+++ ^down^	+ ^down^
*deps-1::gfp(ax2063)*	*gfp::deps-1(ax2063); mut-2(ne298)*	*mut-2*	GFP-DEPS-1	+++^down^	+++ ^down^	−
*deps-1::gfp(ax2063)*	*gfp::deps-1(ax2063); prg-1(n4357)*	*prg-1*	GFP-DEPS-1	+^up^	+++ ^down;^*	+++^up^
*deps-1::gfp(ax2063)*	*gfp::deps-1(ax2063); mut-15(tm1358)*	*mut-15*	PRG-1	−	−	−
*deps-1::gfp(ax2063)*	*gfp::deps-1(ax2063); mut-16(pk710)*	*mut-16*	PRG-1	−	+ ^down^	−
*deps-1::gfp(ax2063)*	*gfp::deps-1(ax2063); mut-2(ne298)*	*mut-2*	PRG-1	−	−*	−
*mut-16(pk710); mut-16::gfp(mgSi2 IV)*	*mut-16(pk710); mut-16::gfp(mgSi2 IV); deps-1(bn121)*	*deps-1*	PRG-1	++^up^	−	−
*deps-1::rfp(ax2063); mut-16 (pk710); mut-16::gfp(mgSi2 IV)*	*deps-1ΔPBS::rfp(mj605); mut-16(pk710); mut-16::gfp(mgSi2 IV)*	*deps-1*^*ΔPBS*^	PRG-1	+^up^	+ ^down^	++^up^
*mut-16(pk710); mut-16::gfp(mgSi2 IV)*	*mut-16(pk710); mut-16::gfp(mgSi2 IV); deps-1(bn121)*	*deps-1*	GFP-MUT-16	+^up^	−*	−*
*deps-1::rfp(ax2063); mut-16 (pk710); mut-16::gfp(mgSi2 IV)*	*deps-1ΔPBS::rfp(mj605); mut-16(pk710); mut-16::gfp(mgSi2 IV)*	*deps-1*^*ΔPBS*^	GFP-MUT-16	+^up;^*	−	−*
*mut-16(pk710); mut-16::gfp(mgSi2 IV)*	*mut-16(pk710); mut-16::gfp(mgSi2 IV); prg-1(n4357)*	*prg-1*	GFP-MUT-16	++^down;^*	−*	+ ^down;^*

Two-sided *t*-tests were performed unless marked with * for which Kolmogorov–Smirnov tests were performed. +++ = 0.001 > *p* values; ++ = 0.01 > *p* values > 0.001; + = 0.05 > *p* values; − = *p* values >0.05. ^down^ = Decreased and ^up^= Increased in values compared with corresponding wild-type animals. Source data are provided as a Source Data file.

Interruption of *prg-1* leads to mildly brighter peri-nuclear GFP-DEPS-1 condensates (Fig. 3b and Table 1). In contrast, removal of the PBS leads to DEPS-1^∆PBS^ forming fewer condensates and becoming diffused in the cytoplasm (Fig. 2a). This suggests that the PBS mediates the interaction of DEPS-1 with additional P granule components in addition to PRG-1 (see small RNA section). Dimmer MUT-16 condensates were found in *prg-1* mutant (Fig. 3a, bottom panel and Table 1), again even though PRG-1 resides in the P granules.

Mutations in either *mut-16* or *mut-2*, and to a lesser extent *mut-15*, abolish the perinuclear association of DEPS-1^WT^ (Fig. 3b and Table 1). Surprisingly, despite their effects on DEPS-1 position, *mut-16*, *mut-2* and *mut-15* mutations do not affect the intensity of PRG-1 condensates (Fig. 3d). This indicates that the perinuclear localisation of PRG-1 is not dependent on the presence of DEPS-1 in the same condensate and is in agreement with the more intense PRG-1 condensate localised to the perinuclear region in *deps-1* mutants. Interestingly, we found that in some instances where the brightness of the condensates is affected the circularity and area are also altered (Table 1). These changes may reflect the rearrangements of the small clusters within the condensate. Overall, we found that mutations in *deps-1* and *prg-1* affect MUT-16 condensate (Fig. 3a, e and Table 1). Similarly, mutations in *mutator* genes lead to defects in DEPS-1 condensate (Fig. 3b, c and Table 1). These observations suggest that piRNA pathway proteins located in P granules and mutator foci may be linked.

### *deps-1* is required for 22Gs against some piRNA targets

**Fig. 4:**
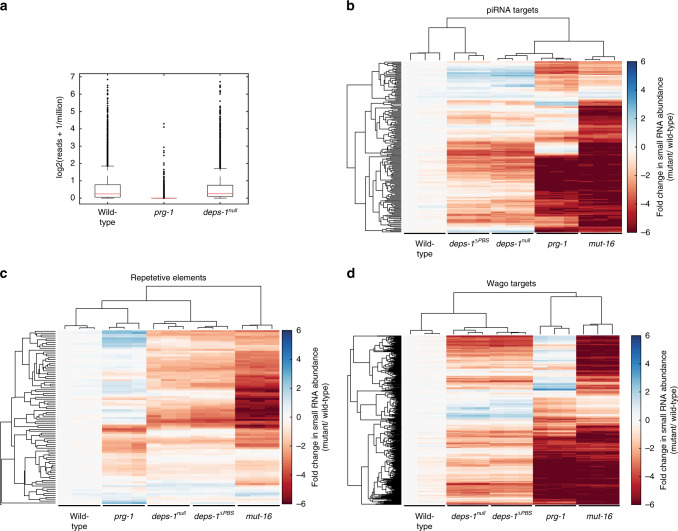
*deps-1* regulates 22Gs against piRNA targets and other endo-siRNAs. **a** Small RNAs were sequenced in animals containing piRNA sensor (*mjIs144*; denoted as wild-type) alone, or in the presence of *deps-1(bn124)* or *prg-1(n4357)* mutations. *deps-1* mutant expresses similar level of 21Us as in wild-type animals, whereas 21Us in *prg-1(n4357)* mutant is significantly diminished compared with wild type and *deps-1* mutant (one-sided *t*-test, *p* value < 10^−20^). *n* = 2 biologically independent samples. Centre line indicates the median, outer boxes represent the 25th and 75th percentiles, whiskers indicate the distance 1.5 times distance between the 25th and 75th percentiles or are limited to the most extreme observation. Outliers are marked if they are greater or less than the whiskers. Cluster analysis of 5’-independent small RNA libraries showing the fold change of small RNAs mapped to known targets of different small RNA pathways: piRNA targets (**b**), repetitive elements (**c**) and *wago* targets (**d**), in the indicated mutants compared to wild type. Wild-type denotes animals expressing *piRNA sensor*(*mjIs144); deps-1*^*null*^ denotes animals expressing *deps-1(bn121)*; *piRNA sensor*(*mjIs144); deps-1*^*ΔPBS*^ denotes animals expressing *deps-1*^*ΔPBS*^*(mj605); mut-16(pk710); mut-16::gfp(mgSi2); mut-16* denotes animals expressing *mut-16*(pk710); *piRNA sensor*(*mjIs144)*. Fold change is displayed in natural log. *n* = 3 biologically independent samples. Source data are provided as a Source Data file.

Having observed that *deps-1* mutations desilence the piRNA sensor, we analysed the small RNA populations in *deps-1* mutants. We first examined the effects of the *deps-1*^*null*^*(bn124)* mutant on piRNA abundance. We sequenced the small RNA population from 5’- independent libraries and show that the *deps-1* mutant has a comparable level of 21U population as in *wild-type* animals (Fig. 4a). We then examined the abundance of secondary siRNAs (22Gs). We found 2012 genes with over 50 reads per million on average across all samples. We observed a significant overlap of 259 genes with greater than twofold reduction in 22Gs levels in *deps-1* mutants (447 genes) and *prg-1* mutants (704 genes; Hypergeometric Test: *p* < 10^−29^; Supplementary Fig. 6a). In addition, we examined the effect of *deps-1* on a published list of piRNA targets^25^, of which 173 exceeded the 50 reads per million threshold, and observed an enrichment for piRNA targets having a > twofold reduction in 22Gs levels (106 out of 447 genes) in *deps-1* mutants (Hypergeometric Test: *p* < 10^−30^; Fig. 4b and Supplementary Fig. 6b). This indicates that *deps-1* is required for the accumulation of 22Gs on a subset of *prg-1* targets.

### *deps-1* functions in multiple small RNA pathways

We next asked if other small RNA populations are affected in *deps-1* mutants. We observed an overlap of 428 genes between the genes that show greater than twofold reduction in secondary siRNA levels in *deps-1* mutants (447 out of 2012 genes) and *mut-16* mutants (761 out of 2012 genes; Hypergeometric Test: *p* < 10^−194^; Supplementary Fig. 6a). Similarly, we found 31 repetitive elements with greater than twofold reduction in *deps-1* mutants (31 out of 2012 genes) which also show greater than twofold reduction in *mut-16* mutants (64 out of 2012 genes; Hypergeometric Test: *p* < 10^−06^; Fig. 4c and Supplementary Fig. 6c). *deps-1* also affects *wago* targets^44^ (300 out of 425 *wago* targets show > twofold reduction in 22Gs level in *deps-1* mutant; Hypergeometric Test: *p* < 10^−^^139^; Fig. 4d and Supplementary Fig. 6d). However, unlike *mut-16* in which 22 out of 23 *ergo-1* targeting 22Gs have > twofold reduction (Hypergeometric Test: p < 10^−08^), *deps-1* has limited effects on *ergo-1* targets^45^ (7 out of 23 genes; Hypergeometric Test: *p* < 0.2; Supplementary Fig. 6e). Lastly, *deps-1* does not affect the 22Gs of *csr-1* targets because we observed a significant reduction in the number of overlapping genes between the *csr-1* targets^46,47^ (4 out of 162 genes) and genes with >twofold reduction in secondary siRNA levels in *deps-1* mutants (447 out of 2012 genes; Hypergeometric Test: *p* < 10^−13^; Supplementary Fig. 6f). Therefore *deps-1* functions in multiple (but not all) germline small RNA pathways which suggests the possibility that *deps-1* might interact with other Ago proteins.

### Small RNAs target P granule-associated genes

**Fig. 5:**
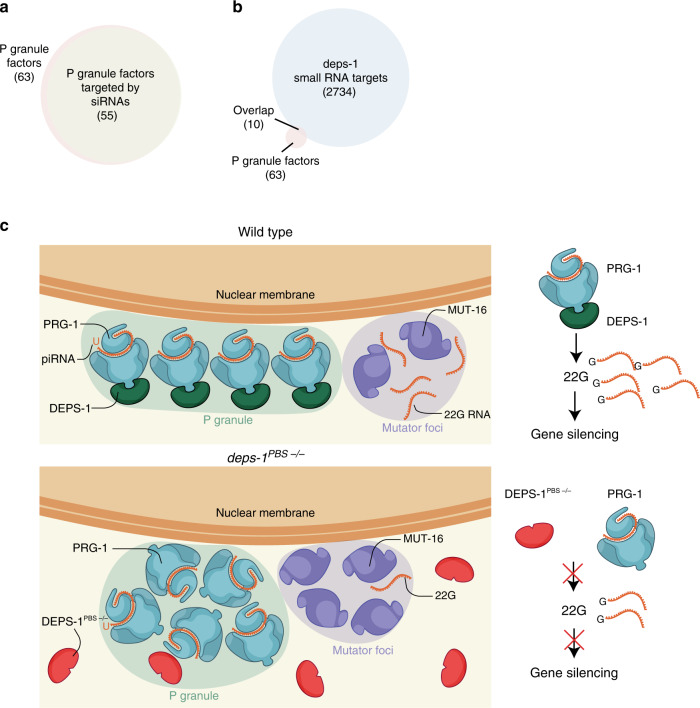
P granule components are targeted by small RNAs and mechanistic model of DEPS-1 function in small RNA pathways. **a** 55 P granule factors are targeted by endo-siRNAs. **b** The 22Gs of 10P granule factors are differentially regulated in *deps-1(bn121)* mutant. **c** Wild-type DEPS-1 binds directly to the PIWI domain of PRG-1 via the PBS motif in the N-terminal. The PRG-1 and DEPS-1 clusters interact to form elongated perinuclear condensates. Intact PRG-1/DEPS-1 complex maintains normal morphology of PRG-1 and MUT-16 condensates. Efficient gene silencing mediated by the piRNA pathway ensues (top panel). DEPS-1 PBS mutant cannot associate with PRG-1 in perinuclear condensates and becomes dispersed in the cytoplasm. This results in an increase in the intensity of PRG-1 and MUT-16 condensates. Additionally, the steady-state level of secondary endo-siRNA from multiple pathways is reduced in the presence DEPS-1 PBS mutant. In particular, the piRNA pathway cannot mediate gene silencing (bottom panel). This figure was created by Claudia Flandoli.

Having observed changes in secondary endo-siRNAs in *deps-1* mutants, we asked whether changes in small RNA in our data correlate with previously published changes in mRNA expression^32^. Spike et al. show that in a *deps-1* mutant, mRNAs of 13 genes are significantly downregulated and 32 genes are significantly upregulated, respectively^32^. We identified that 14 of these genes with different mRNA expression also have altered abundance of targeting small RNA in *deps-1* mutants (Hypergeometric Test: *p* < 10^−06^; Supplementary Fig. 6g). In addition, we found in general when small RNAs are decreased, their target mRNAs are more likely to be upregulated (*R*^2^ = 0.58; One-sample *t*-test *p* < 0.01; Fig. 5a) in *deps-1 null* mutant and vice versa (One-sample *t*-test *p* < 0.1), suggesting the previously described effects on mRNA are mediated by the perturbations in small RNA populations. It remains to be determined which Argonuates are responsible for the changes in mRNAs and 22Gs given *deps-1* is involved in multiple small RNA pathways.

As most germline-expressed genes are targeted by endo-siRNA, it follows that P granule proteins may also be targeted by endo-siRNAs^48,49^ (8986 genes out of 11,088 genes expressed in germline are targeted by endo-siRNA). We obtained a list of P granule factors from AmiGO under the GO term ‘P granule’ and manually curated the list to remove protein isoforms of the same gene. Comparing the P granule list with known endo-siRNA targets of various small RNA pathways, we observed that 55 out of 63P granule factors are endo-siRNA targets (Fig. 5a and Supplementary Data 2) and that 10 out of 63 P granule factors are whose 22Gs are differentially regulated in *deps-1* mutant (Fig. 5b). As expected, P granule factors are not more likely to be targeted by endo-siRNA than other germline expressed genes in general (Hypergeometric Test: *p* < 0.5) or affected in *deps-1* mutant (Hypergeometric Test: *p* < 0.1). Spike et al. show that RDE-4, a protein essential for RNAi, is downregulated in *deps-1* null mutant which is likely the cause of *deps-1* mutants being RNAi resistant^32^. Hence, some of the effect of *deps-*1 mutations on small RNA functions could be indirect and that *deps-1* has a regulatory role on proteins with a direct role in these small RNA pathways.

## Discussion

Small RNA pathways associate with membraneless organelles. Here we reveal a role for the P granule factor DEPS-1 in the piRNA pathway, functioning as a link between piRNAs and secondary endo-siRNAs. Specifically, DEPS-1 directly binds PRG-1 through a conserved PIWI binding site (PBS) regulating 22Gs homoeostasis. Furthermore, the direct interaction with DEPS-1 is important for the organisation of PRG-1 sub-organelle clusters within the P granule (Fig. 5c).

It has long been known that various small RNA machineries, such as Argonaute proteins, localise to P granules. However, it is only recently that a role for the P granule in ensuring appropriate small RNA-mediated silencing has been shown. Using mutant animals that lack P granules in the *C. elegans* primordial germ cells, these studies elegantly show that P granules are required for transgenerational inheritance of RNAi^44,50,51^. The direct interaction between PRG-1 and DEPS-1 provides an example for how small RNAs and P granules can be biochemically coupled.

The piRNA pathway is dependent on protein factors reside both in P granules and mutator foci. Despite PRG-1 forming aberrant condensates in the *deps-1* null mutant, the piRNA population is normal in these animals suggesting that 21Us are still able to associate with PRG-1^24,26^. In contrast, *deps-1* mutations reduce endo-siRNAs of piRNAs origin and lead to brighter and fewer MUT-16 foci. The dependence on *deps-1* for the normal level of 22Gs, but not 21Us, against piRNA targets places *deps-1* downstream of *prg-1*. Therefore, DEPS-1 acts as a functional bridge between P granules and mutator foci. The *C.elegans* germline expresses several small RNA-associated Argonaute proteins that are not localised to the mutator foci, even though the function of these small RNA pathways depend on intact mutator foci^29,30^. The factors that bridge these Argonautes and mutator foci have yet to be fully characterised. ZNFX-1 is one such protein as it has been shown to associate with Argonaute proteins and a RdRP^12^.

Here we observe *deps-1* affects secondary siRNAs of many small RNA pathways, arguing for its function as a facilitator of multiple Argonautes. The PBS motif on DEPS-1 may be capable of recognising the universally conserved PIWI domain of several Argonautes^36,37,52^. Proteins capable of associating with multiple Argonaute proteins, as in the case of ZNFX-1, lead to balanced epigenetic signals. The possibility that DEPS-1 is critical in fine-tuning multiple piRNA and endo-siRNA pathways merits further study.

We have identified EDG-1 as an interacting partner of DEPS-1 and PRG-1. Interestingly, knockdown of *edg-1* specifically affects DEPS-1 condensates and not PRG-1 or the constitutive P granule protein PGL-1. Whether and how *edg-1* regulate small RNA pathways remain to be determined. Indeed, whether EDG-1 is localised to P granules requires further investigation.

Perinuclear germ granules are conserved features throughout the animal kingdom, and are sites of RNA metabolism and RNA-mediated gene regulation^9,53^. The liquid-like property of membraneless organelles is thought to facilitate dynamic internal rearrangements as well as exchange of materials with their surroundings. Recently, a non-dynamic, gel-like scaffold has been found to envelope the liquid-core of P granules^54^; under electron microscopy, the crest and the base of P granules show distinct staining intensity^55^; differences in translational activity between the periphery and the core of the P-bodies have been observed in *Drosophila* oocytes^56^. These suggest subdomains exist within membraneless organelles to support or as a result of their functional complexities. We observe here that PRG-1 and DEPS-1 condensates are formed from smaller clusters of proteins that intertwine. Whether this organisation of PRG-1 and DEPS-1 substructures within the P granule is the result or reflective of the piRNA pathway activity is unclear. In this respect, Wan et al. show that ZNFX-1 forms a condensate that areas appose to both the P granules and Mutator foci^11^, while Ishidate et al. demonstrated that ZNFX-1 interacts with Ago proteins and promotes the Mutator foci machinery to position to the 3’ end of mRNAs^12^. Together, their studies provide evidence that there is a correlation between zones of intense molecular activities and protein localisation.

While much focus has been placed on how proteins drive phase-transition in RNP foci formation, a flurry of recent studies investigated the importance of RNAs in the formation of RNP foci. Langdon et al. show that the secondary structure of mRNAs plays essential roles in specifying distinct Whi3-containing RNPs^57^. Furthermore, RNA:protein ratios determine phase-transition events in proteins prone to solid aggregation^58^. Given that a myriad of small RNA pathways are routed through the P granules and mutator foci in *C. elegans*^59^, it will be important to decipher how the various RNA species contribute to the formation of these organelles.

## Methods

### Immunoprecipitation for mass spectrometry

Synchronised wild-type N2 and *prg-1(n4357)* animals were grown to 1 day-old adults at 20 °C on HB101. After washing thoroughly to remove bacteria, animals were resuspended in lysis buffer (20 mM HEPES, pH7.5, 150 mM NaCl, 0.5% NP-40) and snap frozen in liquid nitrogen. The samples were then lysed by bead-beating, followed by centrifugation at 4 °C to remove insoluble debris. Anti-PRG-1 antibody (Custom) or rabbit IgG was pre-coupled to protein A/G matrix (Thermo Scientific, 88802) and incubated with the supernatant of worm lysates for 4 h (four biological replicates of Anti-PRG-1 with N2 lysates, three biological replicates of anti-PRG-1 with *prg-1(n4357)* lysates and three biological replicates of anti-IgG with N2 lysates). The immunoprecipitants were then washed with 3 × 1 ml of lysis buffer and eluted in elution buffer (8 M urea, 10 mM HEPES pH 8.0) with shaking at room temperature for 30 min.

### LS-MS/MS

Briefly, proteins eluted from immunoprecipitations were reduced and alkylated. Quantified proteins were then digested consecutively in solution using Lys-C and trypsin (both at 1:50 enzyme:substrate ratio)^60^. Peptides were desalted, dried and redissovled in 5% formic acid. RPLC was performed using a Dionex RSLC nano HPLC (Thermo Scientific). Peptides were injected onto a 75 μm × 2 cm PepMap-C18 pre-column and resolved on a 75 μm × 50 cm RP-C18 EASY-Spray temperature-controlled integrated column-emitter (Thermo Scientific) using a 4-h multistep gradient from 5% B to 35% B with a constant flow of 200 nl min^−1^ as described previously. The mobile phases were: 2% acetonitrile (ACN) incorporating 0.1% formic acid (FA) (solvent A) and 80% ACN incorporating 0.1% FA (solvent B). The spray was initiated by applying 2.5 kV to the EASY-Spray emitter and the data were acquired on a Q-Exactive Orbitrap plus (Thermo Scientific) under the control of Xcalibur software in a data-dependent mode selecting the 15 most intense ions for HCD–MS/MS. The survey scan was acquired over an *m*/*z* range 350–1600 with a 70,000 resolution, AGC target of 1e6 ions and a maximum IT of 20 ms. The subsequent MS2 scans were acquired over an *m/z* range of 200–200 *m/z* at 17,500 resolution, an AGC target of 1e5 and 60 ms maximum IT. Peptide ions were isolated with 1.4 Th precursor ion isolation window and fragmented using HCD with normalised collision energy (NCE) of 27. Data have been deposited in PRIDE (project accession code: PXD016838).

### Mass spectrometry data analysis

Raw MS data were processed by MaxQuant^61^. iBAQ values were divided by the total sum of intensity of each sample^62^. These normalised values were then log_10_ transformed to obtain normality and the resulting values were used for student’s *t*-test. To identify proteins enriched in immunoprecipitated PRG-1 from wild-type animals, the medians of the transformed values were used for fold-change calculations.

### Molecular cloning and recombinant protein expression

All PRG-1 and DEPS-1 constructs were cloned using restriction enzymes into the pMAL-C5X vector. Recombinant proteins were expressed in BL21 (DE3) at 37 °C. Briefly, 10 ml of overnight pre-cultures were inoculated to 1 L of LB. Cells were grown to OD_600_ ~0.8 and 1 mM IPTG was added to induce protein expression for 4 h. To purify the proteins, bacterial cells were lysed by sonication in PBS supplemented with protease inhibitors. 20 µg/ml RNaseA was added to the cleared lysates and incubated with gentle rotation overnight. Lysates were then applied to amylose resins (NEB, E8021) and washed with 20 column volumes of binding buffer supplemented with 14 mM beta-mercaptoethanol. Proteins were eluted with binding buffer supplemented with 20 mM maltose and 14 mM beta-mercaptoethanol. Proteins eluted from affinity column were subjected to size-exclusion chromatography equilibrated in assay buffer (30 mM HEPES pH 7.5, 100 mM K-Ac, 2 mM Mg-Ac, 14 mM BME). Proteins used for MST assays were >85% in purity.

### Protein labelling

100 µM of purified proteins were incubated with 1 mM Atto fluor dye for 2 h in the dark. Free dye was subsequently separated from labelled proteins using a G-25 desalting column.

### Peptide sequences

DPES-1 peptide: IPLKFGEVILWNESDCDHDK

FGFR2 peptide: PDFSSQPAVHKLTKRIP

### Microscale thermophoresis (MST)

Proteins/peptides were labelled with atto-488 NHS ester (Sigma, 41698). Free dye was separated from labelled protein using G25 desalting columns. Unlabelled proteins were serial diluted 1:2 and incubated with a constant amount of labelled protein. MST assays were carried in assay buffer supplemented with 0.01% NP-40. Fluorescence was monitored throughout the assay (5 s laser off, 30 s laser on, 5 s laser off). The apparent dissociation constant (*K*_d_^app^) was calculated by the law of mass action using data from either thermophoresis or thermophoresis with temperature jump.

### Small RNA library preparation

Synchronised animals were grown to 1 day-old adults 20 °C. After being washed with M9 to remove bacteria, animals were resuspended in TRIsure (Bioline, BIO-38033). Animals were lysed with 5× freeze-thaw cycles in liquid nitrogen. Total RNA was isolated by chloroform extraction. For 5′ -independent libraries, 5 µg of total RNA was treated with 5′ polyphosphatase (Epicenter, RP8092H). Small RNAs were indexed using the TruSeq small RNA sample kit (Illumina) and size selected by gel separation in 6% TBE gels (Life Tech) and subsequently purified.

### Small RNA analysis

Small RNA sequencing results were obtained from https://basespace.illumina.com/ as fastq files after demultiplexing. Sequencing data is available in the European Nucleotide Archive under study accession number PRJEB31348 (Table Data 4). 3′ Adapter, reads below 18 nt length and reads with a length above 32 were removed using cutadapt. Remaining reads were aligned using STAR against the *C. elegans* genome WS235 allowing multimapping reads. To detect piRNAs, reads of 5′ dependent libraries were mapped against piRNA annotation WS235. Next, piRNA reads were counted using featurecount and abundance was calculated by correcting for library size using unique mapping reads. To compare piRNA sensor read distribution, reads of 5′ independent libraries were mapped against the piRNA sensor using STAR. Small RNA abundance was calculated by correcting for library size using H2B mapping reads. To compare small RNA changes in between worm strains of different gene set, small RNA reads per gene of a specific gene set were counted and abundance calculated by correcting for library size using unique mapping reads (cutoff > 50 reads per million). The mean small RNA abundance per gene was calculated, next the fold-change was calculated by divided the mean abundance in *mutant* animals by the mean abundance in wild-type animals. Gene sets were obtained from Bagijn et al. for piRNA targets^25^, Gu et al. for soma, germline and wago^63^, Claycomb et al. for csr-1^47^, Conine et al. for alg-3/4^46^, Vasale et al. for ergo-1^45^, Buckley et al. for hrde-1^64^ and repetitive element genes were detected using RepeatMasker.

### Worm dissection and immunostaining

1 day-old adult animals were dissected for germline and freeze cracked on poly-lysine coated microscope slides. Dissected germlines were fixed in −20 °C methanol for 20 min. Fixed samples were washed with PBS-T (PBS supplemented with 1% tween-20) prior to primary antibody addition. Primary antibodies were incubated with the samples at 4 °C for overnight. Secondary antibodies were incubated at 37 °C for 1 h in the dark. Antibodies used: anti-PRG-1 (Custom, 1:1000); anti-mouse GFP (Thermofisher, A-11120; 1:400); OIC1D4 for PGL-1 staining (Developmental Studies Hybridoma Bank; 1:50); anti-RFP (Chromotek, clone 5F8). All fluorescence secondary antibodies were from Invitrogen and used at 1:500. Dissected and stained germlines were mounted with Vectorshield antifading agent supplemented with DAPI.

### Confocal microscopy

Images were taken on Leica SP8 confocal microscope. Images taken for granule quantification were of single slices, with pinhole set at 1 AU. HyVolution images were taken with pinhole narrowed to 0.5 AU to result in higher resolution. HyVolution images were deconvoluted using the HyVolution software. Some images were taken on the Zeiss LSM880 using the Airyscan mode.

### Granule pipeline for confocal image analysis

We noticed that the morphology of these perinuclear granules changes, moreover progressively in a transgenerational manner (Supplementary Fig. 6h), we therefore restricted our characterisation to the first five generations post-introduction of these mutations (i.e. the first generation being the first generation when the mutation of interest is homozygous after the cross). To carry out image analysis, we created an analytical pipeline consisting of a general-purpose object segmentation plugin (HKM Segment, https://github.com/gurdon-institute/HKM-Segment) for ImageJ^65^, called by a macro (https://github.com/gurdon-institute/HKM-Segment/blob/master/Kin_granules.ijm) to detect granules and measure intensity, area and circularity in our piRNA-condensate paradigm (Table 1). HKM Segment is a plugin for ImageJ inspired by Alexandre Dufour’s Hierarchical K-Means segmentation algorithm^66^ available in Icy^67^. In this implementation, agglomerative K-Means clustering is applied to the image histogram to determine K threshold levels. An initial set of $K_0$ intensity levels are initially assigned evenly spaced through the intensity range $r = i_{max} - i_{min}$ regardless of frequency, and the merging distance $m = \frac{{i_{max} - i_{min}}}{{K_a}}$ is recalculated for each iteration (a). Clustering is continued until assignment converges to give no further change in levels or cluster assignments, with $2 \le K_a \le K_0$. $K_0$ is therefore the maximum permitted number of clusters and can be set as high as necessary to ensure separation of useful intensity classes, although increasing starting values will converge to the same final $K_a$ when $\frac{{i_{max} - i_{min}}}{{K_a}} \, < \, m$.

The calculated threshold levels are applied in ascending order to extract objects within the specified size range. A thresholding algorithm can be chosen to filter out objects of low intensity, giving robust results in biological images without requiring subsequent level-sets segmentation as used by Dufour et al. The fragments extracted by this method are further clustered to reconstruct objects that remain within the specified size range.

We used a custom ImageJ macro to call HKM Segment and output the results together with additional analysis. Regions of interest created by HKM Segment are output to the ImageJ Roi Manager when it is run from a macro, giving flexibility in the downstream analysis applied to segmented images. In this case, our macro measures the area, circularity and distance from the nearest nucleus boundary for granules detected with HKM Segment. The parameters used were a starting K of 16, a radius range of 0.1–0.6 µm and the Otsu thresholding method^68^.

Images were first manually curated to measure only areas containing germline, complete nuclei and nuclei at the widest cross section before measurements were performed using our macro.

Comparisons between different strains were usually obtained from three biological replicates, 2–4 germlines per replicate from 15 dissected germlines. Control and test strains were dissected and imaged in one setting. To calculate significant differences, mean values of each germline were obtained and tested for normality using the Shapiro–Wilk test. Two-sided Student’s *T*-tests were performed on normally distributed data. Kolmogorov–Smirnov tests, a non-parametric text, were performed on data not normally distributed (which is mostly MUT-16 quantification). For fold change calculations, average intensity of all controls of one experiment was obtained and used to calculate the fold change of the individual mutant germline within the same experiment.

### Western blotting

Proteins from 75–150 µg of worm lysates were resolved by SDS-PAGE and transferred onto PVDF membrane. Antibodies used: Antibodies used: anti-PRG-1 (Custom^69^, 1:1000); anti-tubulin (Sigma, DM1A; 1:1000); anti-DEPS-1 (custom, kind gifts of Strome lab, 1:50).

### RNAi

Adult animals were bleached to obtain embryos which then hatched and synchronised in M9 for 24−48 h at 20 °C. L1 animals were fed with bacteria expressing control dsRNA or dsRNA against *edg-1*. 1-day old adults were subsequently dissected for germline imaging.

### General animal maintenance

Animals were fed with HB101 and maintained at 20 °C (unless stated otherwise) on NGM plates. Strains used in this study are listed in Supplementary Data 3.

### Reporting summary

Further information on research design is available in the Nature Research Reporting Summary linked to this article.

## Supplementary information

Supplementary Information

Reporting Summary

Description of Additional Supplementary Files

Supplementary Data 1

Supplementary Data 2

Supplementary Data 3

Supplementary Data 4

## Data Availability

Data that support the findings of this study have been deposited in PRIDE with the project accession code PXD016838 and European Nucleotide Archive under study accession number PRJEB31348. The source data underlying Figs. 2b, f, 3c–e, 4a–d, 5a and Supplementary Figs. S1a, d, S3b–e, S3g, S5a–d, S6a–f and Table S1. All data is available from the corresponding author upon reasonable request. Source data are provided with this paper.

## Source data

Source Data

## Code availability

Small RNA sequencing analysis code can be accessed via https://github.com/fbnbraukmann/DEPS1_2019. ImageJ plugin can be accessed via https://github.com/gurdon-institute/HKM-Segment. Source data are provided with this paper.

## References

[1] Shin Y, Brangwynne CP (2017). Liquid phase condensation in cell physiology and disease. Science.

[2] Boeynaems S (2018). Protein phase separation: a new phase in cell biology. Trends Cell Biol..

[3] Banani SF, Lee HO, Hyman AA, Rosen MK (2017). Biomolecular condensates: organizers of cellular biochemistry. Nat. Rev. Mol. Cell Biol..

[4] Feric M (2016). Coexisting liquid phases underlie nucleolar subcompartments. Cell.

[5] Molliex A (2015). Phase separation by low complexity domains promotes stress granule assembly and drives pathological fibrillization. Cell.

[6] Hubstenberger A (2017). P-body purification reveals the condensation of repressed mRNA regulons. Mol. Cell.

[7] Meister G (2013). Argonaute proteins: functional insights and emerging roles. Nat. Rev. Genet..

[8] Eulalio A, Behm-Ansmant I, Izaurralde E (2007). P bodies: at the crossroads of post-transcriptional pathways. Nat. Rev. Mol. Cell Biol..

[9] Voronina, E., Seydoux, G., Sassone-Corsi, P. & Nagamori, I. RNA granules in germ cells. *Cold Spring Harb. Perspect. Biol*. **3**, a002774 (2011). 10.1101/cshperspect.a002774 PMC3225947 21768607

[10] Sheu-Gruttadauria J, MacRae IJ (2018). Phase transitions in the assembly and function of human miRISC. Cell.

[11] Wan G (2018). Spatiotemporal regulation of liquid-like condensates in epigenetic inheritance. Nature.

[12] Ishidate T (2018). ZNFX-1 functions within perinuclear nuage to balance epigenetic signals. Mol. Cell.

[13] Malone CD, Hannon GJ (2009). Small RNAs as guardians of the genome. Cell.

[14] Weick E-M, Miska E (2014). a. piRNAs: from biogenesis to function. Development.

[15] Czech B (2018). piRNA-guided genome defense: from biogenesis to silencing. Annu. Rev. Genet..

[16] Deng W, Lin H (2002). miwi, a murine homolog of piwi, encodes a cytoplasmic protein essential for spermatogenesis. Dev. Cell.

[17] Carmell MA (2007). MIWI2 is essential for spermatogenesis and repression of transposons in the mouse male germline. Dev. Cell.

[18] Kuramochi-Miyagawa S (2004). Mili, a mammalian member of piwi family gene, is essential for spermatogenesis. Development.

[19] Simon M (2014). Article reduced insulin/IGF-1 signaling restores germ cell immortality to *Caenorhabditis elegans* Piwi mutants. Cell Rep..

[20] Gou LT (2017). Ubiquitination-deficient mutations in human piwi cause male infertility by impairing histone-to-protamine exchange during spermiogenesis. Cell.

[21] Ruby JG (2006). Large-scale sequencing reveals 21U-RNAs and additional microRNAs and endogenous siRNAs in *C. elegans*. Cell.

[22] Kamminga LM (2012). Differential impact of the HEN1 homolog HENN-1 on 21U and 26G RNAs in the germline of Caenorhabditis elegans. PLoS Genet..

[23] Montgomery, T. A. et al. PIWI associated siRNAs and piRNAs specifically require the *Caenorhabditis elegans* HEN1 ortholog henn-1. *PLoS Genet*. **8**, e1002616 (2012). 10.1371/journal.pgen.1002616 PMC3334881 22536158

[24] Batista PJ (2008). PRG-1 and 21U-RNAs interact to form the piRNA complex required for fertility in *C. elegans*. Mol. Cell.

[25] Bagijn, M. P. et al. Function, targets, and evolution of *Caenorhabditis elegans* piRNAs. *Science* **337**, 574–578 (2012). 10.1126/science.1220952 PMC3951736 22700655

[26] Das PP (2008). Piwi and piRNAs act upstream of an endogenous siRNA pathway to suppress Tc3 transposon mobility in the *Caenorhabditis elegans* germline. Mol. Cell.

[27] Ashe A (2012). piRNAs can trigger a multigenerational epigenetic memory in the germline of *C. elegans*. Cell.

[28] Shirayama M (2012). PiRNAs initiate an epigenetic memory of nonself RNA in the *C. elegans* germline. Cell.

[29] Zhang C (2011). mut-16 and other mutator class genes modulate 22G and 26G siRNA pathways in *Caenorhabditis elegans*. Proc. Natl Acad. Sci. USA.

[30] Phillips CM, Montgomery TA, Breen PC, Ruvkun G (2012). MUT-16 promotes formation of perinuclear Mutator foci required for RNA silencing in the *C. elegans* germline. Genes Dev..

[31] Uebel CJ (2018). Distinct regions of the intrinsically disordered protein MUT-16 mediate assembly of a small RNA amplification complex and promote phase separation of mutator foci. PLoS Genet..

[32] Spike CA, Bader J, Reinke V, Strome S (2008). DEPS-1 promotes P-granule assembly and RNA interference in *C. elegans* germ cells. Develop..

[33] Houri-Ze’evi L (2016). A tunable mechanism determines the duration of the transgenerational small RNA inheritance in C. *elegans*. Cell.

[34] Paix A (2014). Scalable and versatile genome editing using linear DNAs with microhomology to Cas9 sites in *Caenorhabditis elegans*. Genetics.

[35] Gambarotto D (2018). Imaging cellular ultrastructures using expansion microscopy (U-ExM). Nat. Methods.

[36] Elkayam E (2017). Multivalent recruitment of human argonaute by GW182. Mol. Cell.

[37] Matsumoto N (2016). Crystal structure of silkworm PIWI-clade argonaute Siwi bound to piRNA. Cell.

[38] Takimoto K, Wakiyama M, Yokoyama S (2009). Mammalian GW182 contains multiple Argonaute-binding sites and functions in microRNA-mediated translational repression. RNA.

[39] Pfaff J (2013). Structural features of Argonaute—GW182 protein interactions. Proc. Natl Acad. Sci. USA.

[40] Eulalio A, Behm-Ansmant I, Schweizer D, Izaurralde E (2007). P-body formation is a consequence, not the cause, of RNA-mediated gene silencing. Mol. Cell. Biol..

[41] Bounedjah O (2012). Macromolecular crowding regulates assembly of mRNA stress granules after osmotic stress. J. Biol. Chem..

[42] Phillip Y, Sherman E, Haran G, Schreiber G (2009). Common crowding agents have only a small effect on protein-protein interactions. Biophys. J..

[43] Hubstenberger A, Cameron C, Noble SL, Keenan S, Evans TC (2015). Modifiers of solid RNP granules control normal RNP dynamics and mRNA activity in early development. J. Cell Biol..

[44] Dodson AE, Kennedy S (2019). Germ granules coordinate RNA-based epigenetic inheritance pathways. Dev. Cell.

[45] Vasale JJ (2010). Sequential rounds of RNA-dependent RNA transcription drive endogenous small-RNA biogenesis in the ERGO-1/Argonaute pathway. Proc. Natl Acad. Sci. USA.

[46] Conine CC (2010). Argonautes ALG-3 and ALG-4 are required for spermatogenesis-specific 26G-RNAs and thermotolerant sperm in *Caenorhabditis elegans*. Proc. Natl Acad. Sci. USA.

[47] Claycomb JM (2009). The argonaute CSR-1 and its 22G-RNA cofactors are required for holocentric chromosome segregation. Cell.

[48] Bezler A (2019). Tissue- and sex-specific small RNAomes reveal sex differences in response to the environment. PLoS Genet..

[49] West SM (2018). Developmental dynamics of gene expression and alternative polyadenylation in the *Caenorhabditis elegans* germline. Genome Biol..

[50] Lev I (2019). Germ granules govern small RNA inheritance. Curr. Biol..

[51] Ouyang, J. P. T. et al. P granules protect RNA interference genes from silencing by piRNAs. 10.1101/707562 (2019). 10.1016/j.devcel.2019.07.026 PMC6764750 31402283

[52] Schirle NT, MacRae IJ (2012). The crystal structure of human Argonaute2. Science.

[53] Updike DL, Hachey SJ, Kreher J, Strome S (2011). P granules extend the nuclear pore complex environment in the *C*. *elegans* germ line. J. Cell Biol..

[54] Putnam, A., Cassani, M., Smith, J. & Seydoux, G. A gel phase promotes condensation of liquid P granules in *Caenorhabditis elegans* embryos. *Nat. Struct. Mol. Biol*. **26**, 220–226 (2019). 10.1038/s41594-019-0193-2 PMC6668929 30833787

[55] Sheth U, Pitt J, Dennis S, Priess JR (2010). Perinuclear P granules are the principal sites of mRNA export in adult *C*. *elegans* germ cells. Development.

[56] Weil TT (2012). Drosophila patterning is established by differential association of mRNAs with P bodies. Nat. Cell Biol..

[57] Langdon, E. M. et al. mRNA structure determines specificity of a polyQ-driven phase separation. *Science* (80-). 10.1126/science.aar7432 (2018). 10.1126/science.aar7432 PMC6192030 29650703

[58] Maharana, S. et al. RNA buffers the phase separation behavior of prion-like RNA binding proteins. *Science* (80-). eaar7366 10.1126/science.aar7366 (2018). 10.1126/science.aar7366 PMC6091854 29650702

[59] Grishok, A. Biology and mechanisms of short RNAs in *Caenorhabditis elegans*. *Adv. Genet.* 1–69 10.1016/B978-0-12-407675-4.00001-8 (2013). 10.1016/B978-0-12-407675-4.00001-8 23890211

[60] Bensaddek D (2016). Micro-proteomics with iterative data analysis: Proteome analysis in *C. elegans* at the single worm level. Proteomics.

[61] Cox J, Mann M (2008). MaxQuant enables high peptide identification rates, individualized p.p.b.-range mass accuracies and proteome-wide protein quantification. Nat. Biotechnol..

[62] Schwanhäusser B (2011). Global quantification of mammalian gene expression control. Nature.

[63] Gu W (2009). Distinct argonaute-mediated 22G-RNA pathways direct genome surveillance in the *C. elegans* germline. Mol. Cell.

[64] Buckley B (2012). a. et al. A nuclear Argonaute promotes multigenerational epigenetic inheritance and germline immortality. Nature.

[65] Rasband, W. S. Image J, U. S. National Institutes of Health, Bethesda, Maryland, USA. http://imagej.nih.gov/ij/ (2014).

[66] Dufour, A., Meas-Yedid, V., Grassart, A. & Olivo-Marin, J. C. Automated quantification of cell endocytosis using active contours and wavelets. In *Proc. International Conference on Pattern Recognition*. 1–4 10.1109/ICPR.2008.4761748 (2008).

[67] De Chaumont F (2012). Icy: An open bioimage informatics platform for extended reproducible research. Nat. Methods.

[68] Otsu N (1979). A threshold selection method from gray-level histograms. IEEE Trans. Syst. Man. Cybern..

[69] Weick EM (2014). PRDE-1 is a nuclear factor essential for the biogenesis of Ruby motif-dependent piRNAs in *C. elegans*. Genes Dev..

