# Supplementary material for: DEPS-1 is required for piRNA-dependent silencing and PIWI condensate organisation in Caenorhabditis elegans
Source: Nat Commun. 2020 Aug 25;11:4242. doi: 10.1038/s41467-020-18089-1 (PMC7447803; doi:10.1038/s41467-020-18089-1)
Supplement: Supplementary file 1 — Supplementary Information [file 41467_2020_18089_MOESM1_ESM.pdf]

Supplementary Information

DEPS-1 is required for piRNA-dependent silencing and PIWI condensate organisation in *Caenorhabditis elegans*

Suen, Braukmann et al.

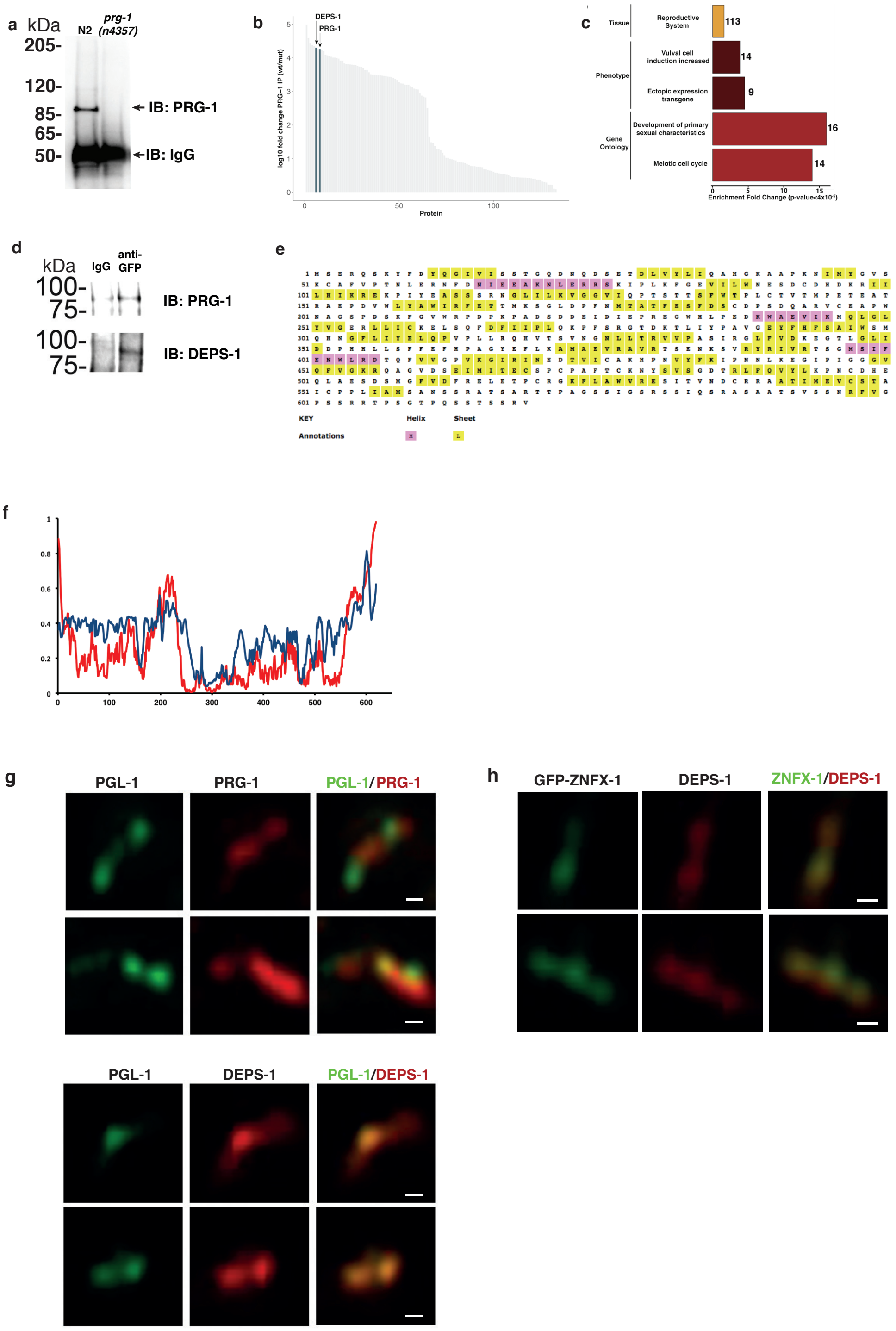

Supplementary Figure 1 DEPS-1 forms a complex with PRG-1.

a) PRG-1 immunoprecipitation for mass spectrometry analysis. Anti-PRG-1 antibody immobilised on Protein A/G magnetic beads was incubated with lysates from wild-type or

prg-1(n4357) animals. Western blot analysis of IPs shows PRG-1 was successfully pulldown. n=3 of independent experiment. Source data are provided as a Source Data file.

b) Proteins co-immunoprecipitated with endogenous PRG-1 were identified by LC-MS/MS. Median normalised iBAQ values were used to calculate fold-change enrichment in PRG-1 IP. Proteins enriched in PRG-1 IP with p-values <0.05 are ranked according to fold-change. P-values were determined by 2-sided t-tests.

c) Proteins enriched in PRG-IP with p-values<0.05 and enrichment fold change >1.5 compared to negative controls were analysed for tissue, phenotypic and gene ontology enrichment terms (p-values determined by hypergeometric tests). Number at each bar indicates number of genes contribute to the enriched terms.

d) DEPS-1 was immunoprecipitated using anti-GFP antibody and analysed for the presence of PRG-1 via western blotting (biological replicate = 1). Source data are provided as a Source Data file.

e) and f) *In silico* analysis of DEPS-1 structure. Secondary structure prediction of DEPS-1 by PSIPRED shows DEPS-1 is rich in β-sheets. Red: α-helix; Yellow: β-sheet. (e) Disorder prediction conducted by IUPRED suggests DEPS-1 does not contain large segments of disordered region (f).

g) Colocalisation of PRG-1 and PGL-1 (upper panel) and DEPS-1 and PGL-1 (bottom panel). Dissected germlines co-stained for DEPS-1 and GFP-PGL-1 were imaged and deconvoluted in Airyscan mode (n=3 germlines). Scale bar = 0.25 µm.

h) Colocalisation of DEPS-1 and ZNFX-1. Dissected germlines co-stained for DEPS-1 and GFP-ZNFX-1 were imaged and deconvoluted in Airyscan mode (n=3 germlines). Scale bar = 0.25 µm.

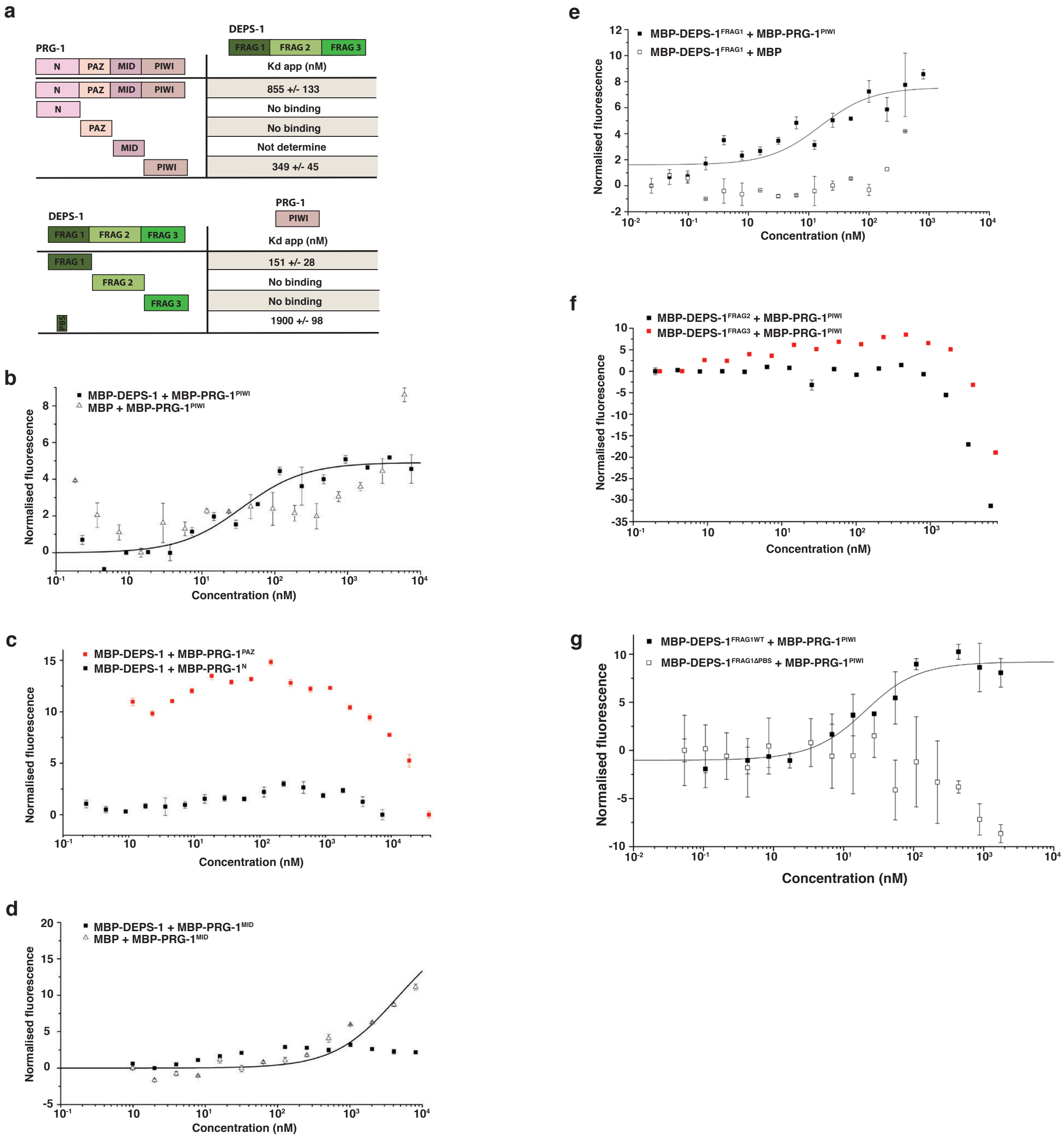

Supplementary Figure 2 PRG-1 and DEPS-1 interact directly.

a) Summary table of K_d^{app} of interactions between full length and truncated domains of DEPS-1 and PRG-1.

b) - d) Full-length MBP-tagged DEPS-1 was fluorescently labelled and tested for binding with unlabeled MBP-tagged PRG-1^{PIWI} (b), PRG-1^{MID} (c), PRG-1^{PAZ} and PRG-1^{N} (d) using MST. The K_d^{app} for binding between full length DEPS-1 and PRG-1PIWI is 349 +/- 45 nM. Representative of n=2 independent experiments. Data are presented as mean values +/- SD of 3 technical replicates.

e) and f) MBP-tagged PRG-1^{PIWI} domain was labelled and tested for interaction with unlabeled MBP- DEPS-1^{frag1} (e), DEPS-1$^{frag\text{-}2}$ or DEPS-1$^{frag\text{-}3}$ (f) using MST. The K_d^{app} for binding between DEPS-1^{frag1} and PRG-1^{PIWI} is 151+/- 28 nM.

g) Unlabeled MBP-PRG-1^{PIWI} domain was tested for binding with labeled wild-type MBP-DEPS-1^{frag1} or MBP- DEPS-1^{frag1} PBS mutant using MST. The K_d^{app} for binding between wild-

type DEPS-1^{frag1} and PRG-1^{PIWI} is 134+/- 28 nM whereas no binding was detected for the DEPS-1^{frag1} PBS mutant.

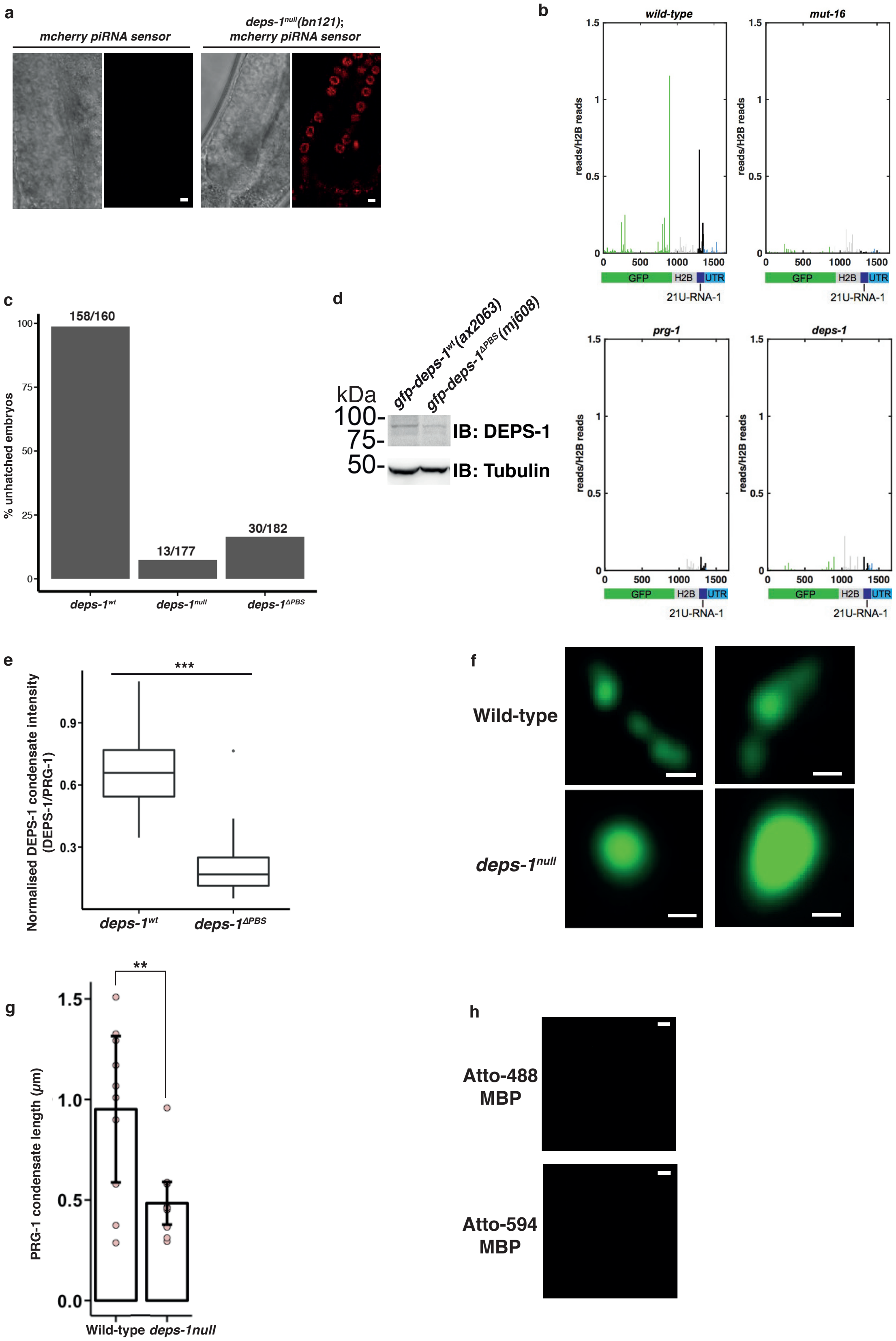

Supplementary Figure 3

a) Mutations in *deps-1* lead to piRNA sensor transgene desilencing. Whitefield (right panel) and fluorescent (left panel) images of whole mounted animals show the mcherry piRNA

sensor is efficiently silenced in wild-type animals and is desilenced *deps-1*^{null} (bn121). Scale bar = 3 µm.
b) Analysis of small RNAs mapped to the piRNA sensor in wild type*, mut-16, prg-1* and *deps-1 mutants*.
c) *deps-1*$^{\Delta PBS}$ animals are germline RNAi incompetent. *pos-1* was targeted by RNAi in animals expressing either *rfp-deps-1*^{WT}, *rfp-deps-1*$^{\Delta PBS}$ mutant or *deps-1*^{null}*(bn121)* in the presence of *gfp-mut-16*; *mut-16(pk710)*. *rfp-deps-1*$^{\Delta PBS}$ mutant animals exhibited similar level of RNAi competency as *deps-1*^{null}*(bn121)* while *rfp-deps-1*^{WT} animals can fully mediate germline RNAi.
d) DEPS-1 protein levels were assessed in *gfp-deps-1*^{wt}*and gfp-deps-1*$^{\Delta PBS}$*(mj608)* mutant animals by western blotting (biological replicate = 1). Source data are provided as a Source Data file.
e) PRG-1 condensates contain less DEPS-1$^{\Delta PBS}$ than wild-type DEPS-1 proteins. Intensity of DEPS-1 was normalized to PRG-1 intensity. Measurements were obtained manually from 23 PRG-1 condensates from 2 germlines (***2-sided t-test p-value=5.4 x10^{-14}). Center line indicates the median, outer boxes represent the 25th and 75th percentiles, whiskers indicate the distance 1.5 times distance between the 25th and 75th percentiles or are limited to the most extreme observation. Outliers are marked if they are greater or less than the whiskers.
f) PRG-1 condensates are malformed in *deps-1*^{null} *(bn121)* mutant. Dissected germlines were stained for PRG-1 were imaged and deconvoluted in Airyscan mode (n=24 condensates per genotype from 2 dissected germlines). Top panels: N2; Bottom panels: *deps-1 (bn121)*. Scale bar = 0.25 µm.
g) The length of PRG-1 condensate along the perinuclear membrane was measured manually. Bar graph represents twenty PRG-1 condensates measured in 1 germline (total n=10 for each genotype). Data presented here as mean values +/- SD. Scale bar = 0.25 µm.
h) 28uM Atto-488 MBP and 21uM Atto-594 MBP were imaged in the presence of 5% PEG2000. No condensates or aggregates were observed (n=3 independent areas). Scar bar = 10 µm.

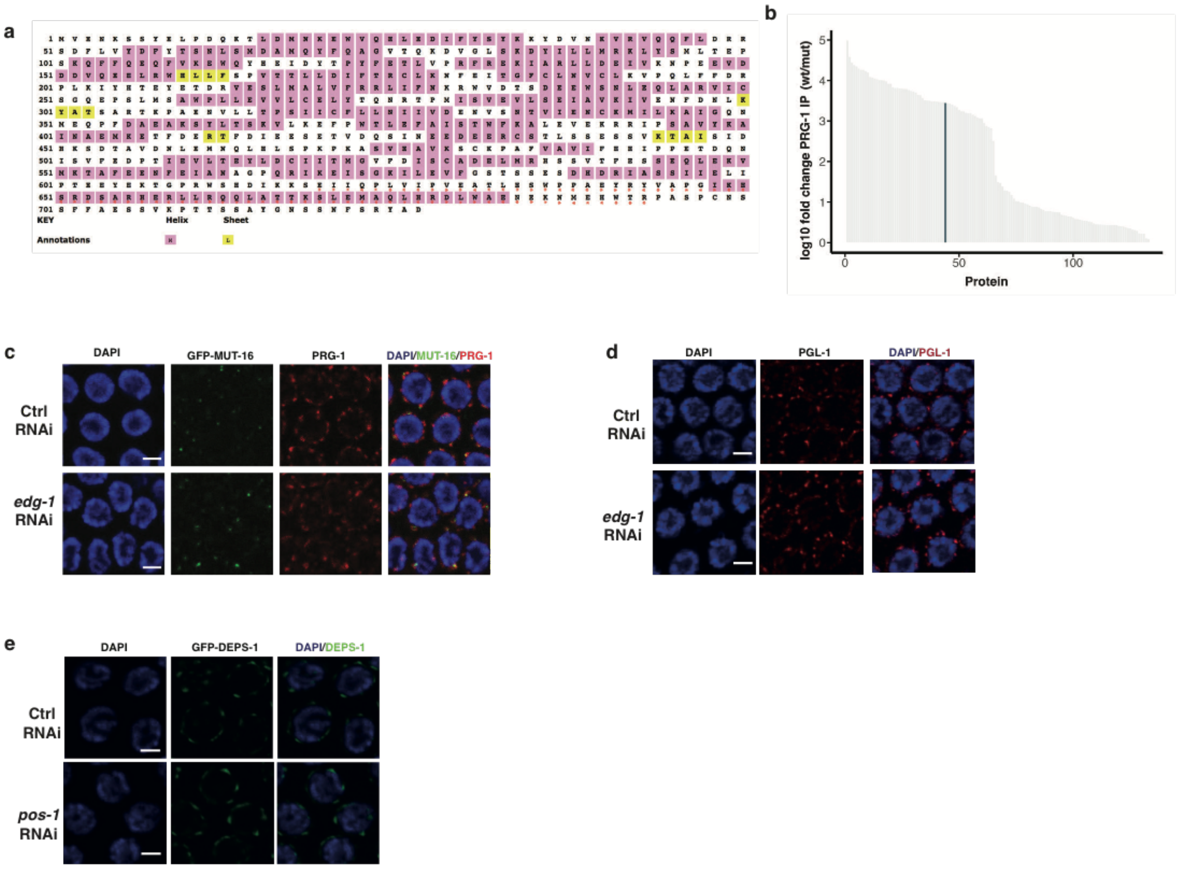

Supplementary Figure 4 EDG-1 interacts with both DEPS-1 and PRG-1.

a) EDG-1 is predicted to be rich in α-helix by PSIPRED and lacks disordered region. Red asterisks highlight the minimal segment required for binding to DEPS-1 in yeast-two-hybrid screen.

b) EDG-1 (highlighted in grey) was enriched in PRG-1 IP as identified by MS/MS. Source data are provided as a Source Data file.

c) Animals expressing gfp::mut-16 (*mut-16 (pk710);mut-16::gfp*(*mgSi2))* were dissected and stained for GFP and PRG-1 after *edg-1* knockdown via RNAi. MUT-16 and PRG-1 granules were not affected by *edg-1* knockdown (n=3 germlines for each condition). Scale bar = 3 µm.

d) N2 animals were dissected and stained for PGL-1 upon *edg-1* knockdown via RNAi. PGL-1 was not affected by *edg-1* knockdown (n=3 germlines for each condition). Scale bar = 3 µm.

e) Animals expressing *gfp::deps-1* (*ax2063)* were dissected and stained for GFP and after *pos-1* knockdown via RNAi. GFP-DEPS-1 condensates are not affected by knockdown of *pos-1* (n=3 germlines for each condition). Scale bar = 3 µm.

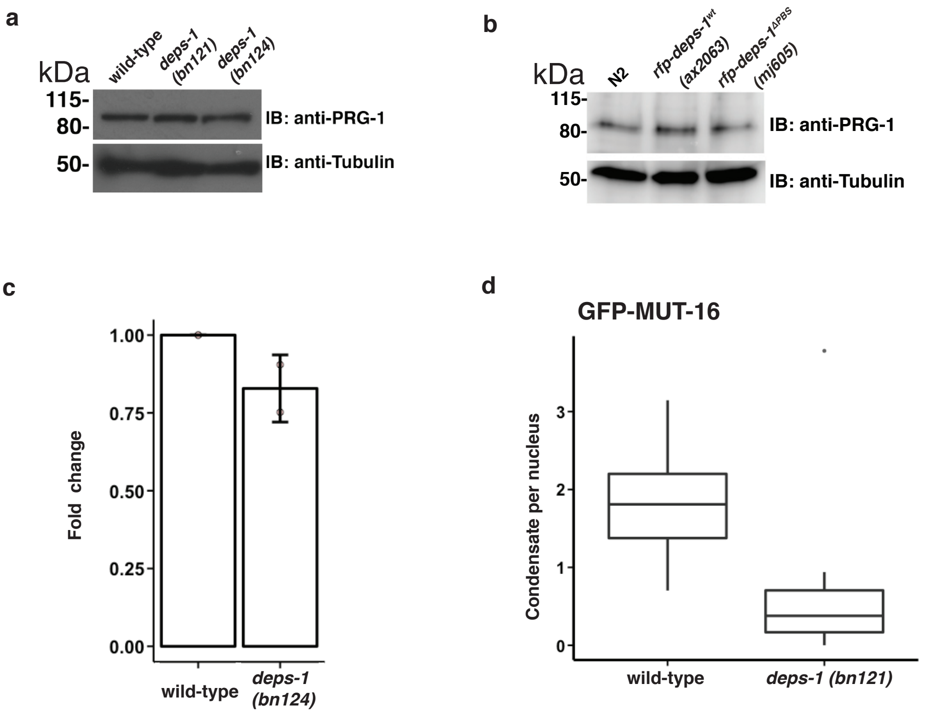

Supplementary Figure 5

a) - c) PRG-1 protein and mRNA levels are not altered in *deps-1* mutant animals.
PRG-1 protein levels were assessed in N2 wild-type*, deps-1(bn121) and deps-1(bn124)* mutant animals (a, representative of 2 biological replicates) and *deps-1(mj608)* animals (b; 1 biological replicate) by western blotting. RT-qPCRs were performed on N2 *wild-type* and *deps-1(bn124)* mutant animals. *prg-1* transcript levels were normalised to *act-3.* Average values of technical replicates of 3 of each biological replicate were used. Error bars are standard deviation of biological replicate of 2 (c). Source data are provided as a Source Data file.
d) The number of GFP-MUT-16 condensate per nucleus in *deps-1 (bn121)* animals is reduced compared to N2 wild-type animals. n=10 germlines. Center line indicates the median, outer boxes represent the 25th and 75th percentiles, whiskers indicate the distance 1.5 times distance between the 25th and 75th percentiles or are limited to the most extreme observation. Outliers are marked if they are greater or less than the whiskers. Source data are provided as a Source Data file.

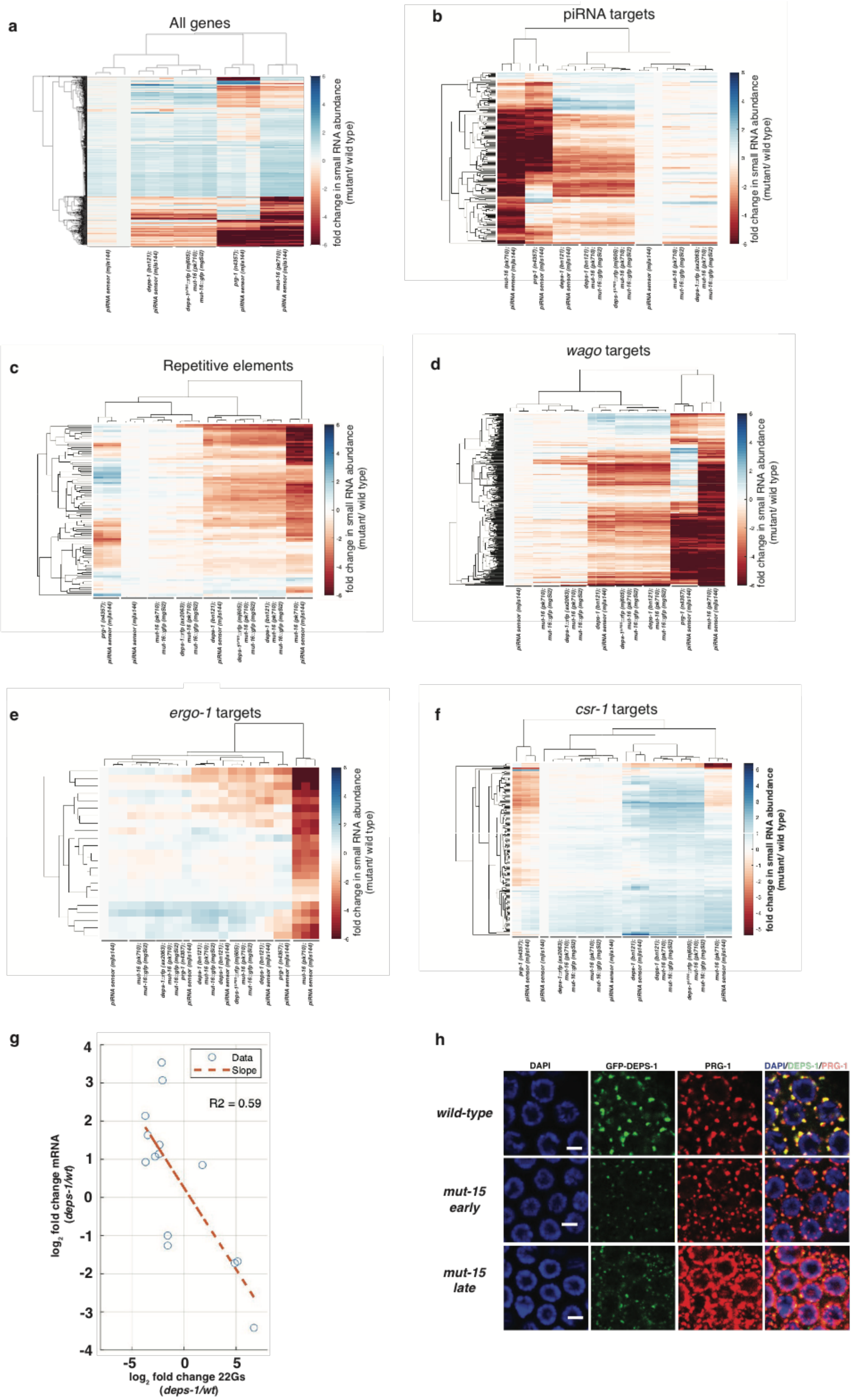
a
All genes
fold change in small RNA abundance (mutant/ wild type)
b
piRNA targets
fold change in small RNA abundance (mutant/ wild type)
c
Repetitive elements
fold change in small RNA abundance (mutant/ wild type)
d
wago targets
fold change in small RNA abundance (mutant/ wild type)
e
ergo-1 targets
fold change in small RNA abundance (mutant/ wild type)
f
csr-1 targets
fold change in small RNA abundance (mutant/ wild type)
g
Data
Slope
R2 = 0.59
log2 fold change mRNA (deps-1/wt)
log2 fold change 22Gs (deps-1/wt)
h
DAPI
GFP-DEPS-1
PRG-1
DAPI/DEPS-1/PRG-1
wild-type
mut-15 early
mut-15 late

Supplementary Figure 6 DEPS-1 affects multiple small RNA pathways.

a) - f) Cluster analysis of small RNA libraries showing the fold change of small RNAs mapped to known targets of different small RNA pathways: all genes (a), piRNA targets (b), repetitive elements (c), wago12 targets (d), *ergo-1* targets (e), *csr-1* targets (f), in the indicated mutants compared to wild type. Fold change is displayed in natural log. Source data are provided as a Source Data file.

g) Fold change in 22Gs and mRNA transcripts of differentially regulated genes in *deps-1* null mutant were compared. Fold changes of mRNA transcript were obtained from Spike et. al., 2008. Differential regulation of 22Gs is defined as adjusted p-value < 0.05 derived from 2-sided t-tests, fold change compared to wild-type either <0.5 or >2. Source data are provided as a Source Data file.

h) Transgenerational effects in PRG-1 and DEPS-1 condensates upon mutations in *mut-15.* Defects in PRG-1 perinuclear accumulation in late generation of *mut-15* mutant. Dissected germlines co-stained for PRG-1 and GFP-DEPS-1 in wild-type and *mut-15*(*tm1358)* mutant animals. wild-type indicates the common genotype of *gfp::deps-1(ax2063)* among the strains used. Accumulation of PRG-1 to the perinuclear region in *mut-15* mutant is comparable to wild-type animals in early generations but is strongly affected in late generations. Micrographs representative of 3 independent experiments. Scale bar = 3 µm.
