# Supplementary material for: DEPS-1 is required for piRNA-dependent silencing and PIWI condensate organisation in Caenorhabditis elegans
Source: Nat Commun. 2020 Aug 25;11:4242. doi: 10.1038/s41467-020-18089-1 (PMC7447803; doi:10.1038/s41467-020-18089-1)
Supplement: Supplementary file 2 — Reporting Summary [file 41467_2020_18089_MOESM2_ESM.pdf]

nature research

Corresponding author(s): Eric A. Miska

Last updated by author(s): 16 Jun 2020

Reporting Summary

Nature Research wishes to improve the reproducibility of the work that we publish. This form provides structure for consistency and transparency in reporting. For further information on Nature Research policies, see Authors & Referees and the Editorial Policy Checklist.

Statistics

For all statistical analyses, confirm that the following items are present in the figure legend, table legend, main text, or Methods section.

n/a	Confirmed	
☐	☒	The exact sample size (n) for each experimental group/condition, given as a discrete number and unit of measurement
☐	☒	A statement on whether measurements were taken from distinct samples or whether the same sample was measured repeatedly
☐	☒	The statistical test(s) used AND whether they are one- or two-sided<br>*Only common tests should be described solely by name; describe more complex techniques in the Methods section.*
☐	☒	A description of all covariates tested
☐	☒	A description of any assumptions or corrections, such as tests of normality and adjustment for multiple comparisons
☐	☒	A full description of the statistical parameters including central tendency (e.g. means) or other basic estimates (e.g. regression coefficient) AND variation (e.g. standard deviation) or associated estimates of uncertainty (e.g. confidence intervals)
☐	☒	For null hypothesis testing, the test statistic (e.g. F, t, r) with confidence intervals, effect sizes, degrees of freedom and P value noted<br>*Give P values as exact values whenever suitable.*
☒	☐	For Bayesian analysis, information on the choice of priors and Markov chain Monte Carlo settings
☒	☐	For hierarchical and complex designs, identification of the appropriate level for tests and full reporting of outcomes
☒	☐	Estimates of effect sizes (e.g. Cohen's d, Pearson's r), indicating how they were calculated

Our web collection on statistics for biologists contains articles on many of the points above.

Software and code

Policy information about availability of computer code

Data collection	HiSeq 1500 was used for RNA Seq data collection; Proteomics data was collected on Q-Exactive Orbitrap in data-dependent mode.
Data analysis	Code repository for small RNA seq data can be found herehttps://github.com/fbnbraukmann/DEPS1_2019, Genome:<br>WB235<br><br>Annotation:<br>WBcel235.78<br><br>Read trimming<br>cutadapt 1.16<br><br>Read alginment<br>STAR STAR_2.5.2a<br>--alignIntronMax 1<br>--outFilterMismatchNmax 0<br>--scoreDelOpen -10000<br>--scoreInsOpen -10000<br>--outFilterMultimapNmax 10000<br>--winAnchorMultimapNmax 50<br>--outMultimapperOrder Random<br>--alignEndsType EndToEnd<br><br>Read counting<br>subread-1.5.2<br>featurecCounts

-M --fraction (countMultiMappingReads fractional count)

Clusteranalysis
Matlab2019 clustergram function
'RowPdist', 'euclidean'
'ColumnPdist', 'euclidean'';

Proteomics data was analysed by MaxQuant followed by R studio 1.0.143.
Super-resolution microscopy data was analysed by Hyvolution software.
Image J plugin can be assessed via https://github.com/gurdon-institute/HKM-Segment

For manuscripts utilizing custom algorithms or software that are central to the research but not yet described in published literature, software must be made available to editors/reviewers. We strongly encourage code deposition in a community repository (e.g. GitHub). See the Nature Research guidelines for submitting code & software for further information.

Data

Policy information about availability of data

All manuscripts must include a data availability statement. This statement should provide the following information, where applicable:

- Accession codes, unique identifiers, or web links for publicly available datasets
- A list of figures that have associated raw data
- A description of any restrictions on data availability

Small RNA Seq data: European Nucleotide Archive under study accession number PRJEB31348 (Figures 4, S2g and S5);
Proteomics data (Figure S3 and S1): PRIDE project accession code PXD016838

Field-specific reporting

Please select the one below that is the best fit for your research. If you are not sure, read the appropriate sections before making your selection.

☒ Life sciences ☐ Behavioural & social sciences ☐ Ecological, evolutionary & environmental sciences

For a reference copy of the document with all sections, see nature.com/documents/nr-reporting-summary-flat.pdf

Life sciences study design

All studies must disclose on these points even when the disclosure is negative.

Sample size	Small RNA Seq data: 3 biological replicates per strain for secondary small RNAs and 2 biological replicates for piRNAs; Proteomics data: 3 biological replicates for the two negative controls and 4 biological replicates for the test sample; germline dissection: 2-3 biological replicates of 2-4 germline randomly imaged out of 15 germlines dissected for each biological replicate; MST data was carried out as 3 technical replicates with 2 independent experiments. Sample sizes were not predetermined by statistical methods but by conventional requirements in the respective fields.
Data exclusions	There were two Images of germline where background signal is too high for the image analysis pipeline (Granule pipeline) to identify the mut-16 condensate accurately and these were excluded.
Replication	All attempts to reproduce data were successful. Please see 'sample size' section for how often replications for each type of experiments were carried out.
Randomization	Randomization was not required because experimental controls were carried out and were not subjective.
Blinding	Blinding not required. The experiments in this study were not subjective.

Reporting for specific materials, systems and methods

We require information from authors about some types of materials, experimental systems and methods used in many studies. Here, indicate whether each material, system or method listed is relevant to your study. If you are not sure if a list item applies to your research, read the appropriate section before selecting a response.

Materials & experimental systems

n/a	Involved in the study
☐	☒ Antibodies
☒	☐ Eukaryotic cell lines
☒	☐ Palaeontology
☐	☒ Animals and other organisms
☒	☐ Human research participants
☒	☐ Clinical data

Methods

n/a	Involved in the study
☒	☐ ChIP-seq
☒	☐ Flow cytometry
☒	☐ MRI-based neuroimaging

Antibodies

Antibodies used	anti-PRG-1 and anti-DEPS-1 are custom antibodies; anti-mouse GFP (Thermofisher, A-11120); OIC1D4 (Developmental Studies Hybridoma Bank). All fluorescence secondary antibodies were from Invitrogen (A28175;A27034;A-11012; ani A-21244;A-21247;A-11007;A-11006;A-11008) . Anti-rabbit HRP-linked antibody from CST, A0545; anti-tubulin from Sigma, clone DM1A; anti-RFP (Chromotek, clone 5F8).
Validation	Anti-PRG-1 was validated using the prg-1 null strain (e.g. figure 3a); anti-mouse GFP was validated using wild-type animals; OIC1D4 was validated by previous studies by other groups e.g. Spike et al 2008. Other commercially available antibodies are validated by the manufacturers and information is available on their websites.

Animals and other organisms

Policy information about studies involving animals; ARRIVE guidelines recommended for reporting animal research

Laboratory animals	C. elegans: strains, all are hermaphrodites:<br>N2 (Bristol)<br>SX3293<br>SX3257<br>SX3323<br>SX3397<br>SX3369<br>GR1799<br>SX3266<br>SX3294<br>SX3327<br>SX3330<br>SX3380<br>SX3370<br>SX1316<br>SX3249<br>SX1888<br>SX1636<br>SX3346<br>DG3226<br>SX922<br>SX3426<br>SX3427<br>YY916
Wild animals	No wild animals were used.
Field-collected samples	Study did not involve field-collected samples.
Ethics oversight	C. elegans studies do not require ethical approval.

Note that full information on the approval of the study protocol must also be provided in the manuscript.
