# Supplementary material for: DEPS-1 is required for piRNA-dependent silencing and PIWI condensate organisation in Caenorhabditis elegans
Source: Nat Commun. 2020 Aug 25;11:4242. doi: 10.1038/s41467-020-18089-1 (PMC7447803; doi:10.1038/s41467-020-18089-1)
Supplement: Supplementary file 3 — Description of Additional Supplementary Files [file 41467_2020_18089_MOESM3_ESM.pdf]

Description of Additional Supplementary Files

File Name: Supplementary Data 1
Description: Putative PRG-1 interacting partners identified by LC-MS/MS. Proteins identified with p-values

File Name: Supplementary Data 2
Description: List of P granule factors targeted by small RNA pathways. T-tests were performed.

File Name: Supplementary Data 3
Description: List of strains used in this study.

File Name: Supplementary Data 4
Description: Sample description of small RNA sequencing data.
